# Machine Learning for Enhanced Identification Probability in RPLC/HRMS Nontargeted Workflows

**DOI:** 10.1021/acs.analchem.5c01873

**Published:** 2025-08-12

**Authors:** Hiu-Lok Ngan, Viktoriia Turkina, Denice van Herwerden, Hong Yan, Zongwei Cai, Saer Samanipour

**Affiliations:** † State Key Laboratory of Environmental and Biological Analysis, Department of Chemistry, 26679 Hong Kong Baptist University, Kowloon, Hong Kong 999077 P. R. China; ‡ Department of Biology, Hong Kong Baptist University, Kowloon, Hong Kong 999077 P. R. China; § Van ’t Hoff Institute for Molecular Sciences (HIMS), 1234 University of Amsterdam, Amsterdam 1098 XH, The Netherlands; ∥ UvA Data Science Center, University of Amsterdam, Amsterdam 1012 WP, The Netherlands

## Abstract

In HRMS-based nontargeted analysis (NTA), spectral matching is crucial for chemical identification, particularly in the absence of retention information. This study introduces class probability of true positives (*P*(*TP*)) as an innovative approach, leveraging data from MS/MS spectra and calibrant-free predicted retention time indices (RTIs) through 3 machine learning (ML) models to enhance identification probability (IP). The first model is a molecular fingerprint (MF)-to-RTI model trained on 4713 calibrants. The second model, a cumulative neutral loss (CNL)-to-RTI model, utilized 485,577 experimental spectra. The final model, a binary classification model, was trained using 1,686,319 *TP* and semisynthetic true negative (*TN*) spectral matches. High correlations between MF-derived and CNL-derived RTI values (*R* ^2^ = 0.96 for training; 0.88 for testing) suggest reduced RTI errors in *TP* spectral matches. Incorporating reference spectral library searches and RTI errors, the k-nearest neighbors algorithm achieved a weighted *F*1 score of 0.65 and a Matthews correlation coefficient of 0.30 for pesticides at concentrations of 1 to 1000 ppb in blank samples, with a recall of 0.60 in black tea matrices. Compared to solely library matching, the average IPs for pesticides increased by 54.5, 52.1, and 46.7% when spiked in blank, 10× diluted, and 100× diluted tea matrices, respectively. This work demonstrates the effectiveness of ML in enhancing the chemical IPs of annotated compounds within complex matrices.

## Graphical abstract

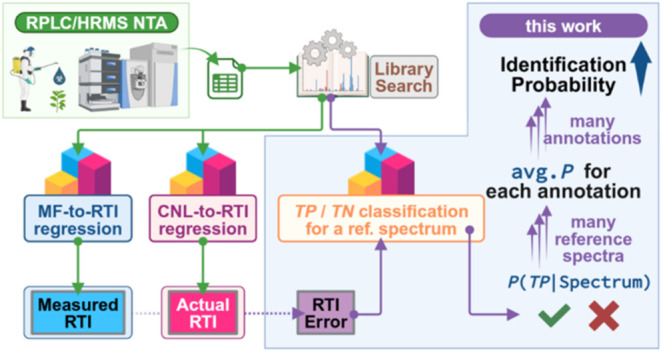

## Introduction

Mass spectrometry (MS)-based nontargeted analysis (NTA) is a high-throughput technique that profiles sample analytes, distinguishing itself from targeted analysis through its discovery-oriented approach. The integration of reversed-phase liquid chromatography (RPLC) with high-resolution MS (HRMS) has emerged as a prominent methodology for analyzing the chemical exposome,^1,2^ including toxic contaminants such as pesticides^3^ and per- and polyfluoroalkyl substances (PFAS).^4^ These contaminants are detected in surface and groundwater, potentially affecting drinking water supplies.^5,6^ Consequently, RPLC/HRMS-based NTA is increasingly employed for screening analyses.

Chemical identification confidence (IC) is necessary for compound annotation in LC/MS-based NTA. The Chemical Analysis Working Group at the Metabolomics Standards Initiative proposed minimum metadata requirements for non-novel metabolite identification in 2007.^7^ For an annotated two-dimensional (2D) LC/MS tensor, IC is considered putative if its mass-to-charge ratio (*m*/*z*) and tandem MS (MS/MS) spectrum match references from external libraries when verification by chemical standards is lacking. Since 2014, similar criteria have been introduced in environmental analysis,^8–10^ emphasizing the necessity for further experimental efforts,^11^ such as matching the retention behavior of reference standards under identical instrumental conditions to enhance confidence.^8^

While in-house databases of standard retention times (RTs) and MS/MS spectra efficiently provide high IC, their establishment requires significant resources. In silico analyses can assist in screening annotated compounds needing further validation, typically involving spectral matching against *m*/*z* values of molecular ions and their fragment ions,^12,13^ followed by ranking candidate hits based on spectral entropy^14^ or similarity.^15,16^ False discovery rate (FDR) is commonly used to adjust the matching scores thresholds, allowing more annotations with lower confidence levels.^14,17^ However, as multiple reference spectra and hits for each incident MS/MS spectrum complicate library searches, a method to assess the overall quality of chemical annotations (considering confidence and ambiguity) is lacking.

Class probability assessments can evaluate the probability of individual true positive (*P*(*TP*)) spectral matches.^18–21^ Recently, the concept of “identification probability” (IP) was introduced by Metz et al.,^22^ advocates for transferable annotation results across analytical platforms, allowing for multiple hits to be considered after calculating the average *P*(*TP*) for each hit. Model transferability—the ability to predict accurately beyond the original training data^23^—is crucial for forecasting chemical retention behavior. This requires diverse data sources and substantial volumes for statistical modeling. Previous studies have reported machine learning (ML) models for RT prediction of the chemical exposome,^24–28^ but their transferability is often limited by varying chromatographic conditions.^29–33^ Although there are different schemes of RT index (RTI), a harmonized RTI scale is necessary to provide a method-independent alternative for the integration with spectral information to enhance IC.^34–37^

This study presents a computational approach for calibrant-free, transferable RTI prediction on a harmonized scale, integrating it with spectral matching for *P*(*TP*) determination and chemical IP enhancement. Our approach employs 3 ML models, including 2 built on quantitative structure-retention relationship (QSRR), to compute expected RTI values for chemicals beyond calibrant data. A binary classification model that incorporates information from both retention and *m*/*z* domains is used to determine *P*(*TP*) for each matched reference spectrum. The average *P*(*TP*) is then calculated for chemical IP determination. We demonstrate our framework using RPLC/HRMS NTA data from pesticides-spiked blanks and black tea samples, aiming to improve nontargeted screening for compounds requiring further validation.

## Materials and Methods

### Overall Roadmap

This study employed 3 ML models alongside the MS/MS spectral matching algorithm, ULSA.^16^ As illustrated in Figures 1 and S01, Model 1 is a random forest (RF) regression model correlating molecular fingerprints (MFs) to true RTI values of calibrants across 3 comparable scales. Model 2 is another RF regression model predicting expected RTI values on a harmonized scale, using experimental MS/MS spectra from various external databases without knowledge of the extract structure. Model 3 is a k-nearest neighbors (KNN) binary classification model calculating *P*(*TP*) for each matched reference spectrum with a matching score of ≥50%. This model integrates features from both RTI and *m*/*z* domains and was validated using independent data sets, including RPLC/HRMS data from pesticide analyses in blank spike and tea extracts.

**Figure 1:**
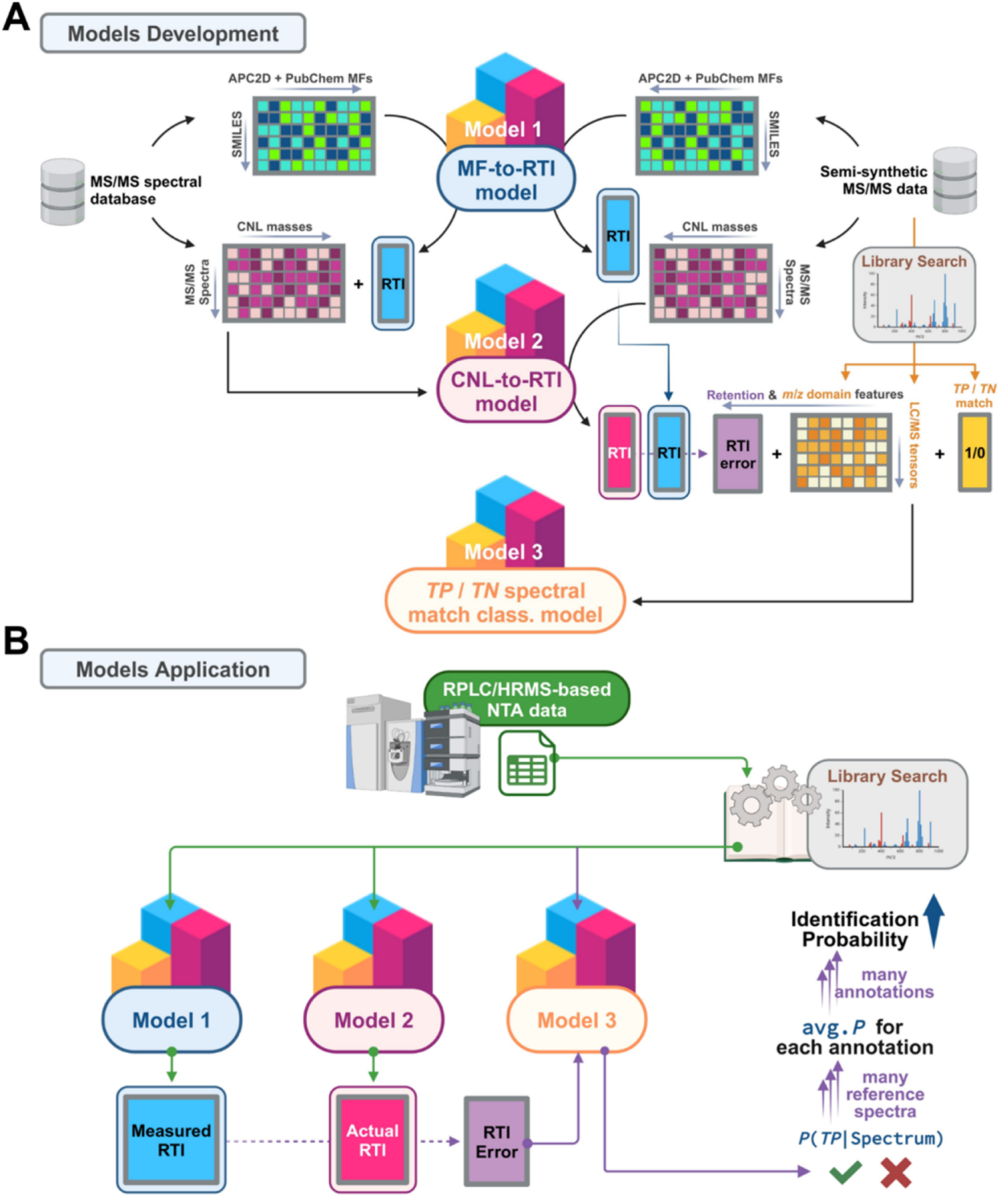
A diagram of the roadmap in this study. (A) Three models were developed in this study. Model 1, a random forest (RF) regression (reg.) model predicting molecular fingerprint (MF)-derived retention time index (RTI). Model 2, a RF reg. model for predicting cumulative neutral loss (CNL)-derived RTI. Semisynthetic MS/MS data were prepared, and Models 1 and 2 predicted expected RTI values for these data, which were processed by library search to output spectral matching scores for training Model 3. Model 3 was a k-Nearest neighbors (KNN) binary classification (class.) model with features from retention and *m*/*z* domains to predict true positive (*TP*) and true negative (*TN*) reference spectral matches. (B) The 3 models were applied to analyze RPLC/HRMS-based nontargeted analysis (NTA) data, enhancing chemical identification probability. *Abbreviations: APC2D, AtomPairs2DFingerprintCount; PubChem, PubChem Fingerprinter*.

### Model 1: Molecular Fingerprint-Based Regression Model

A previously reported RF model for predicting a vector of molecular RTIs (3 scales) was retrained to predict a single RTI on a harmonized scale,^37^ correlating 790 preselected MFs to a RTI (Figure 1A). Unlike previous studies, stratification was based on MFs rather than RTIs to ensure training by structural diversity. This model was trained on 4713 calibrants with simplified molecular-input line-entry system (SMILES) identities across 3 comparable scales in RPLC: the C3–14 *n*-alkylamide system,^38^ the RTI system developed by Aalizadeh et al.,^35^ and the C0–23 cocamide diethanolamine homologous series.^39^ Details on modeling are discussed in Supporting Information. The accuracy of predicted RTI values was assessed using 1237 untrained calibrants, focusing on mean absolute error (MAE) and mean relative error (MRE). The list of compounds used for modeling is available online (see **file 1** at 10.5281/zenodo.16402775).

### Model 2: Cumulative Neutral Loss-Based Regression Model

In addition to predicting expected RTI values from 2D chemical descriptors, a second RF regression model was established to correlate empirical positive electrospray ionization (ESI+) MS/MS data to RTI values predicted by Model 1 using a similar strategy as a prior study (Figure S01).^36^ This model employs cumulative neutral loss (CNL) masses as features, which have proven effective for matching analogous molecules^13,40,41^ and chemical componentization.^13,42^ Based on the previous results,^36,43^ a total of 15,961 high probability masses-of-interest (MOIs) were preselected (the list is available online, see **file 2** at 10.5281/zenodo.16402775), along with monoisotopic mass as an additional feature due to its discriminative power.^36^

The training data set comprised MS/MS reference data from known compounds with InChIKeys and SMILES IDs, totaling 27,211 distinct molecules and 693,685 MS/MS spectra (the list is available online, see **file 3** at 10.5281/zenodo.16402775). Each spectrum contained at least 2 MOIs.^44^ Model 2 was trained on 485,577 query MS/MS spectra. Train/test split were based on CNL leverage. Details on modeling are discussed in Supporting Information. Exploratory data analysis (EDA) and quartile analysis were performed to assess the distributions and the closeness of predicted and true RTI values. Predictive power was evaluated on 208,104 query spectra using root-mean-square error (RMSE, calculated according to eq 1), MRE, and *R* ^2^ value as performance metrics.

### Spectral Reference Library and Universal Library Search Algorithm

ULSA,^16^ a previously developed algorithm, was employed to annotate compounds by matching MS/MS spectra from various reference spectral databases. During ULSA execution, 7 matching parameters were summed to derive a final matching score, as discussed in Supporting Information. These parameters, derived from statistical calculations, can be generated independently of ULSA. Publicly available spectral libraries are integrated with ULSA, allowing for new reference data to be uploaded and matched, which is particularly valuable for annotating emerging chemicals of concern.

### Model 3: Computing *P*(*TP*) for Individual Reference Spectral Match

Model 3 determines whether a spectrum match is a *TP* (labeled “1” as shown in Figure 1A) or true negative (*TN*) (labeled “0”). Features for this model include RTI error (as defined by eq 2) between RTI values derived from Models 1 (*RTI*_*MF*_) and 2 (*RTI*_*CNL*_), monoisotopic mass and 4 parameters obtained from ULSA. A larger RTI error indicates a *TN* spectral match while a smaller error correlates with a *TP* match. Pearson correlation was used to eliminate redundant features with *r* values >0.80 and less importance. Various ML algorithms, including logistic regression (LR), decision tree (DT), RF, and KNN, were evaluated, with a focus on the model’s sensitivity to excluded features. Details on modeling and semisynthetic data preparation are discussed in Supporting Information. A set of *TP* and semisynthetic *TN* MS/MS spectra was analyzed by ULSA, yielding 4,368,902 spectral matches. Matches with scores <50% were classified as *TN*, making ML unnecessary for distinguishing *TP* from *TN*. Ultimately, 1,686,319 spectra were used for training and 421,381 for testing. Among the training samples, 1,535,009 spectra were *TN* (labeled “0”), while 151,310 instances represented *TP* (label “1”). Nine additional replicates of *TP* spectra were included to balance the data set. The optimal model was assessed primarily based on the Matthews correlation coefficient (MCC) score (eq 3) and secondarily by weighted *F*1 score (eq 4) using an external independent data set of pesticides spiked into blank samples at varying concentrations (1, 2.5, 5, 10, 25, 50, 100, and 1000 ppb). For real sample analyses, pesticides were spiked into 10× diluted and 100× diluted black tea matrices. Recall (as defined by eq 5) was chosen as the primary performance metric since *TN* and *FP* rates are unknown in real samples.

### Model Applicability Domain (AD)

Leverage (*h*_*ii*_) was used to assess whether a matched compound fell within the ADs of Model 1 (*h*_*ii*_ < 0.275) and Model 2 (*h*_*ii*_ < 0.146). A leverage threshold was set at the 95% leverage of the model training data, calculated by eq 6 (where *X* represents the matrix of training data, and *x*_*i*_ is the vector for an individual data query).

### Identification Probability Calculation

Multiple compound hits and reference spectra can be matched, yet each spectral match gives a *P*(*TP*). To measure ambiguity in a candidate RPLC/HRMS tensor, an average *P*(*TP*) was calculated for each compound hit (Figures 1B and S01). A decision threshold of 0.50 was applied to determine whether to retain or exclude a hit. IP was then calculated by eq 7 based on the number of shortlisted hits.

### Computations and Code Availability

All scripts for ML modeling and data visualization were written in Julia v.1.6 using Visual Studio Code (Microsoft), with additional visualizations done in Python v.3.10 on Jupyter Notebook (Anaconda3). Details on computational resources and packages are available in Table S1. Updates will be published with the final DOI citation.

## Results and Discussion

### Performance of Model 1 (Molecular Fingerprint-to-Retention Time Index Model)

The first model developed in this study predicted *RTI*_*MF*_ (measured RTI for each suggested match) on a harmonized scale. It was trained using 4713 compound calibrants with 5048 true RTI values across 3 similar RTI scale systems. MAE and MRE of the predicted values for the trained calibrants were 89.52 and 16.23%, respectively. In contrast, the mean absolute difference and the mean relative difference for the calibrants with their true RTI values from 2 different scales were 78.23 and 11.50% (see **file 4** at 10.5281/zenodo.16402775), indicating similar uncertainty for the predicted values from Model 1. When tested against 1263 true values from 1237 calibrants, the uncertainty increased slightly, with a MAE of 111.15 and a MRE of 27.53%. The true RTI values and *CNL*_*MF*_ values exhibited similar distributions in both training (Figure 2A) and testing (Figure 2B) data sets, validating the retrained MF-to-RTI model’s transferability.

**Figure 2:**
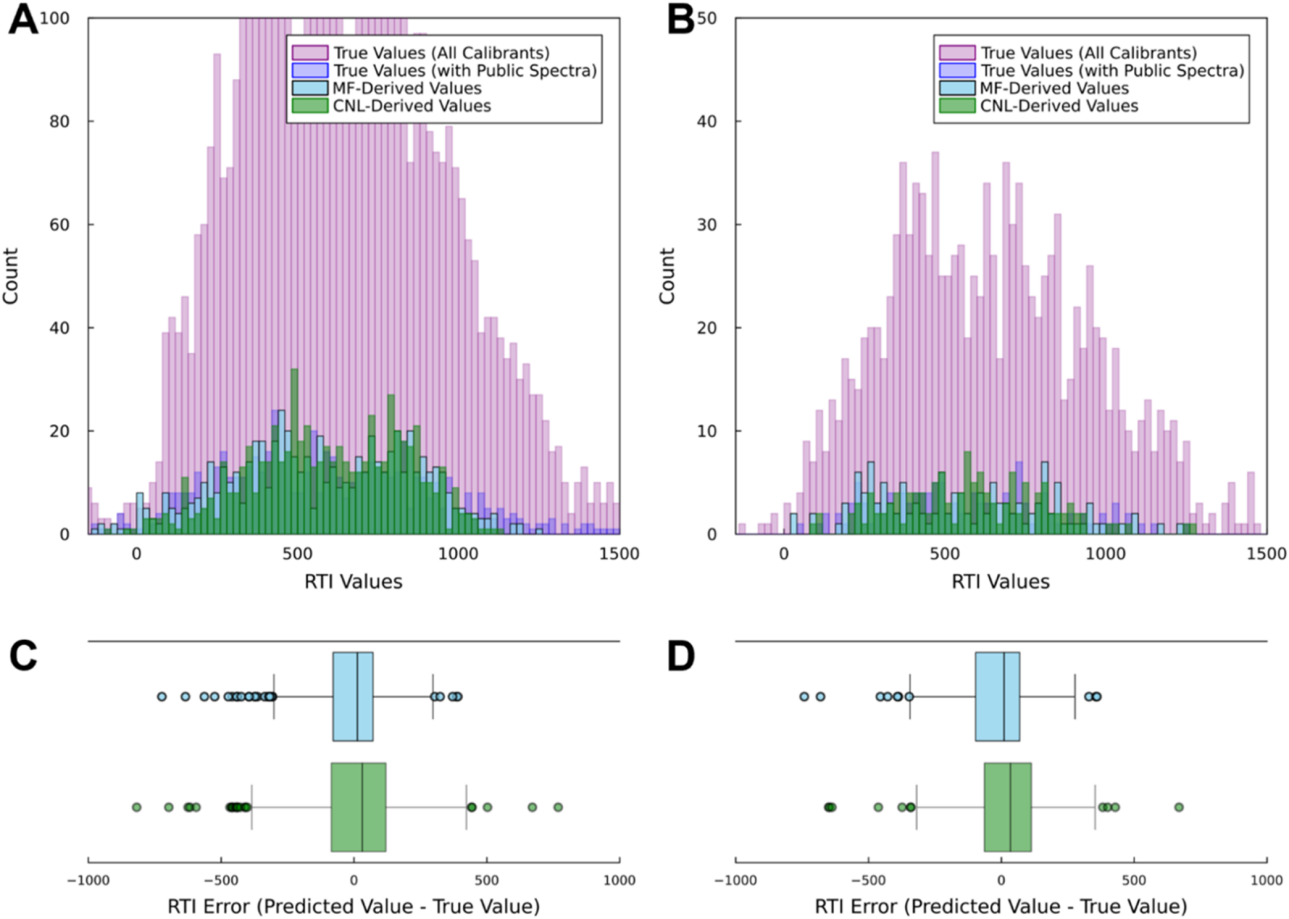
Performance assessment of the quantitative structure-retention relationship (QSRR)-based models. Histograms illustrate the distributions of true retention time indices (RTIs) of calibrants used to (A) train and (B) test Model 1, along with predicted values from Models 1 and 2. Boxplots present the variances of predicted values against true values of calibrants used to (C) train and (D) test Model 1.

### Performance of Model 2 (Cumulative Neutral Loss-to-Retention Time Index Model)

The distribution of *RTI*_*CNL*_ were similar to *RTI*_*MF*_ and the true RTI values of chemical calibrants used to train (Figure 2A) and test (Figure 2B) Model 1. Variances in RTI error from Model 1 was smaller than those from Model 2 (Figure 2C) for the data originally used to train Model 1. However, both models showed similar RTI error variances for extended data not used in Model 1’s training (see Figure 2D). These patterns indicate the exchangeability of RTI values computed from Models 1 and 2.

For the model trained with a 7:3 train/test split, *RTI*_*MF*_ values correlated well with *RTI*_*CNL*_ values in training (Figure S02A) and testing (Figure S02B) data sets. The RMSEs were 51.8 and 92.8, equivalent to MREs of 6.90% and 9.92% with *R* ^2^ values of 0.96 and 0.88 for the training and testing data sets, respectively (Table 1). These results were comparable to the predictive power of the CatBoost model trained on the NORMAN data set using different descriptors by Boelrijk et al.^36^ Our retrained RF model demonstrated better correlation with MF-derived RTIs (train: *R* ^2^ = 0.94; test: 0.85 in Boelrijk et al.’s work). Larger RMSEs were observed in our study (train: 44.0; test: 67.0 in Boelrijk et al.’s work) could be due to the RTI scale adopted across 3 systems. The comparable predictive performance confirms that Model 2 is transferable and effectively predicts expected RTI values based on QSRR.

**Table 1:** Summary of the Machine Learning Models’ Predictive Power

ML^a^ model	model 1	model 2			model 3		
function	to predict RTI^b^ from MF^c^	to predict RTI^b^ from CNL^d^			to predict acceptance/rejection decision of an individual reference spectral match		
performance metric		RMSE^e^	MRE^f^	*R* ^2^	weighted *F*1 score	MCC^g^	recall
training		51.8	6.90%	0.9634	0.89	0.77	0.99
testing		92.8	9.92%	0.8824	0.89	0.77	0.99
validation					0.65	0.30	0.66
real Sample							0.60

a Machine learning.

b Retention time index.

c Molecular fingerprint.

d Cumulative neutral loss.

e Root mean square error.

f Mean relative error.

g Matthews correlation coefficient.

### Performance of Model 3 (*TP*/*TN* Individual Reference Spectral Match Determination)

Model 3, a KNN model incorporated 6 features: “RefMatchFragRatio”, “MS1Error”, “MS2ErrorStd”, “FinalScoreRatio”, monoisotopic mass, and RTI error. It was applied only to individual spectral matches with scores ≥50% for *TP*/*TN* match determination. The model achieved a weighted *F*1 score of 0.65 and a MCC score of 0.30 in bulk analyses of NTA data from pesticides-spiked blank samples at concentrations from 1 to 1000 ppb (Table 1). Notably, the inclusion of RTI error improved recall (0.60) for individual reference spectral matches in 10× and 100× diluted black tea matrices compared to the RTI error-exclusive model (recall = 0.54). This demonstrates that Model 3 achieved acceptable transferability to real samples Figure 3.

**Figure 3:**
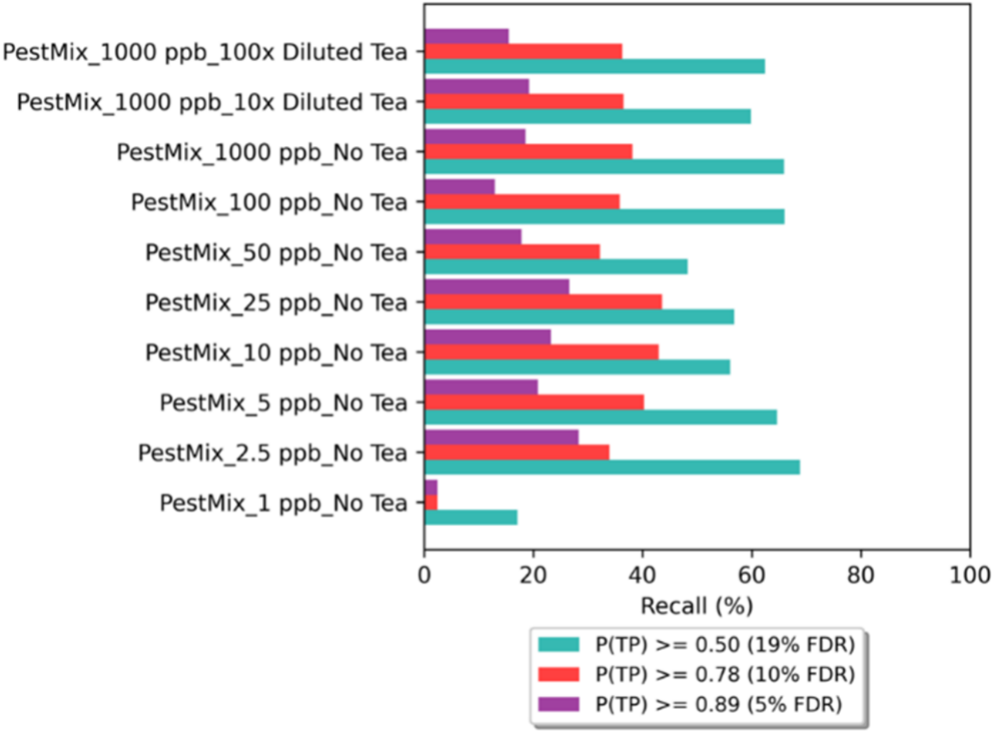
Recalls of pesticide-spiked solutions at different *P*(*TP*) cutoff thresholds. Pesticide standards from the LC/MS Pesticide Comprehensive Mix Kit (PestMix) were spiked into solvent blank and 10× and 100× diluted black tea matrices to create 1 ppm final solutions for analysis. Various final solution concentrations (1, 2.5, 5, 10, 25, 50, 100, and 1000 ppb) were tested for blank spikes (No Tea).

By setting the default probability threshold for accepting a spectral match as *TP* at 0.50 (i.e., *P*(*TP)* ≥ 0.5), we achieved *TP* and *FP* rates of 98.8 and 23.4%, respectively (Figure S03A), resulting in an acceptable FDR of 19.1% for omics studies.^44^ More rigorous FDR-controlled cut-offs for 5 and 10% FDRs corresponded to *P*(*TP)* values of 0.89 and 0.78, respectively (Figure S03B). These decision thresholds were used to calculate recalls for samples in different IC levels. To balance recall and FDR, we selected 0.50 as the *P*(*TP)* cutoff. For 1 ppm samples, recalls increased as the matrix effect decreased. Our results indicated no predictive power for 1 ppb solutions, while stable analysis of chemical contaminants was achieved at concentrations of 2.5 ppb or higher. Since the recall for the 1 ppm sample was significantly below 50%, we estimated the limit of identification by Model 3 to be between 1 and 2.5 ppb.

### Identification Probability Enhancement Comparison

Taking the measurement of ambiguity in candidate compounds into account, we assessed the improvement achieved through the incorporation of ML by evaluating IP using 2 definitions of “hit”. The first definition referred to a compound whose measured spectrum matches a collection of reference spectra. The fungicide Pencycuron (InChIKey: OGYFATSSENRIKG-UHFFFAOYSA-N) was selected as a successful case for discussion due to its structural complexity, which includes a phenylurea moiety, a cyclopentyl ring, and a chlorobenzyl group. For Pencycuron, identification yielded 5 compound hits through conventional matching (Table 2), resulting in an accuracy of 20% (Figure 4). Deploying Models 1, 2, and 3 alongside spectral library searches allowed us to compute *P*(*TP*) for individual matches. Detailed data of all individual matches for Pencycuron can be found in **file 5** at 10.5281/zenodo.16402775. Averaging *P*(*TP*) from 12 spectral matches resulted in values of 0.73, 0.78, and 0.71 for pesticide samples with no tea, 100× diluted matrix, and 10× diluted matrix, respectively (Table 2), indicating a *TP* hit. If the average *P*(*TP*) values of other compound hits were below 0.50, the IP of Pencycuron was considered 100%. However, compound hit B exhibited a moderate average *P*(*TP*) from 16 matches in the samples with no tea and the 10× diluted matrix (Table 2). Although these IPs decreased from 100 to 50%, their values remained higher than the IPs obtained from the ULSA approach (i.e., 20%, Figure 4).

**Figure 4:**
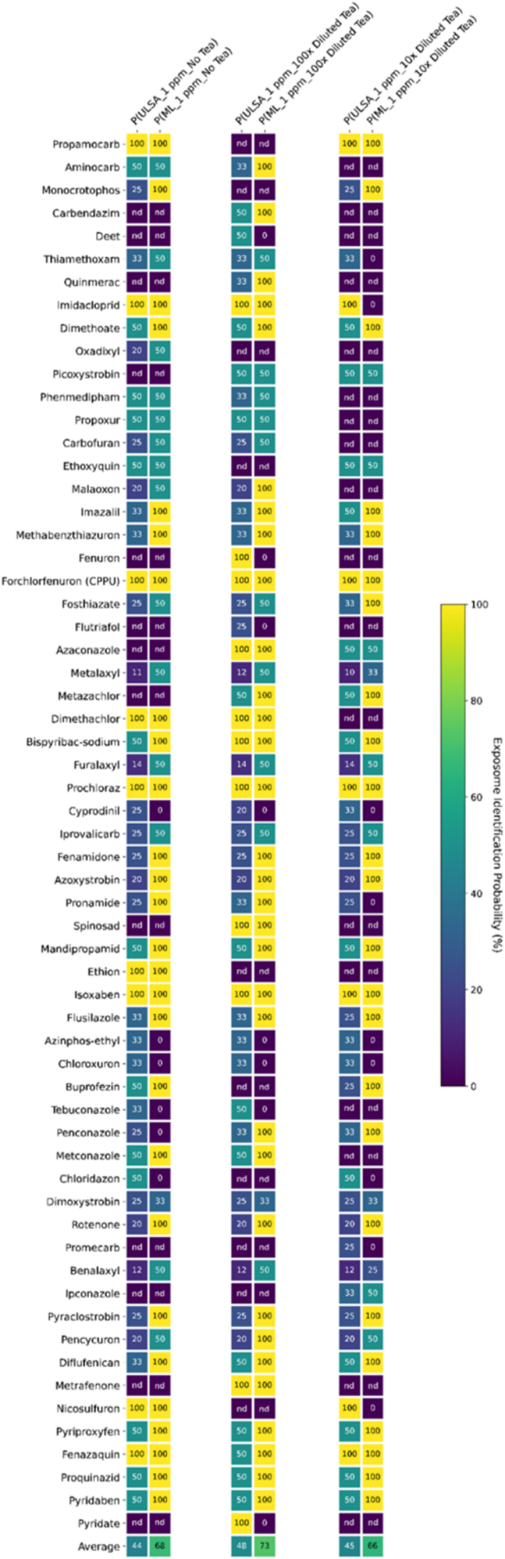
Identification probability (IP) assessment by average *P*(*TP*). Integration of machine learning (ML) with Universal Library Search Algorithm (ULSA) enhanced average IP by 24 and 25% for blank spikes and 100× diluted tea matrix samples, representing increments of 54.5 and 52.1%, respectively. The presence of a 10× diluted matrix reduced this improvement to 21%, equivalent to a 46.7% increase. *Abbreviations: nd, not detected*.

**Table 2:** Summary of the Predictions for 1000 ppb Pencycuron in Various Degrees of Black Tea Matrix

			blank spike			100 times diluted			10 times diluted		
compound	matches	label	prediction	avg. *P*(0)	avg. *P*(1)	prediction	avg. *P*(0)	avg. *P*(1)	prediction	Avg. *P*(0)	Avg. *P*(1)
Hit A^a^ (Pencycuron)	12	1	1	0.27	0.73	1	0.22	0.78	1	0.29	0.71
Hit B^b^	16	0	1	0.43	0.57	0	0.54	0.46	1	0.40	0.60
Hit C^c^	14	0	0	0.60	0.40	0	0.57	0.43	0	0.56	0.44
Hit D^d^	12	0	0	1.00	0.00	0	1.00	0.00	0	1.00	0.00
Hit E^e^	1	0	0	1.00	0.00	0	1.00	0.00	0	1.00	0.00

a InChIKey: OGYFATSSENRIKG-UHFFFAOYSA-N.

b InChIKey: JTVPZMFULRWINT-UHFFFAOYSA-N.

c InChIKey: VCKAUONIDRWIGP-UHFFFAOYSA-N.

d InChIKey: SGUAFYQXFOLMHL-UHFFFAOYSA-N.

e InChIKey: ZDVGWHJBNGTLKF-UHFFFAOYSA-N.

We averaged the IPs of all positive results from our pesticide panel for overall performance comparison. For 1 ppm sample solutions, integrating our KNN model (Model 3) enhanced average IPs from 44 to 68% for blank spikes and from 48 to 73% in the 100× diluted tea matrix samples (Figure 4), representing increments of 54.5 and 52.1%, respectively. The presence of a more concentrated matrix (10× diluted) slightly affected the improvement from ML incorporation, yielding a 21% higher IP than using ULSA alone (from 45 to 66%), which corresponds to a 46.7% increase.

Standard library searches typically rely on ranking individual matches by score.^16,45^ This can mislead the structural assignment results, as only 1 match from multiple reference spectra with the highest score (as known as Top-1 search) is considered, while results from remaining matches with lower scores are neglected. To illustrate this issue, we compared performance by defining a hit as a compound whose measured spectrum matches an individual reference spectrum with high rank that sorted by matching score “FinalScoreRatio” in the ULSA approach, or by individual *P*(*TP*) in the ML-aided approach. An alternative IP, defined in eq 8 especially for ranking analysis, represented the occurrence frequency of *TP* spectral matches among the top-ranked hits. The incorporation of ML for our pesticide panel improved slightly (1–3%) on average for samples with tea matrices, while remaining comparable for spiked blanks (Figure S4). This finding underscores the importance of including as many reference spectra as possible rather than relying on a single reference to account for the identification ambiguity of a candidate compound.^22^

## Conclusions

Integrating ML with reference spectral library searches improved recall in real samples with and without tea matrices and increased annotation confidence compared to single library searches. The application of Model 3 resulted in acceptable weighted *F*1 and MCC scores for the blank spikes, alongside notable IP increases in diluted tea matrices. Greater IP improvements were observed as the number of reference spectra increased. For both tea matrix-containing and matrix-free samples, computing chemical IP from collection of all available reference MS/MS spectra significantly improved annotation confidence, providing a higher confidence in silico analysis solution for early stage data analytics. However, a primary limitation of our approach is the reliance on the quality and diversity of the spectral reference libraries. Incomplete or biased libraries may hinder the identification of highly structurally diverse compounds or those with limited accessible reference data. To address this challenge, continuous updates the reference spectral libraries with denoised spectra and computational spectra are essential. Additionally, ongoing validation with various real-world samples of *TN* MS/MS spectra will be crucial to fine-tuning Model 3.

1 $$\mathrm{RMSE}=\sqrt{\frac{1}{N}\sum_{i=1}^{N}{\left(y_{i}-{ŷ}_{i}\right)}^{2}}$$

2 $$RTI\;error={\mathrm{RTI}}_{\mathrm{MF}}-{\mathrm{RTI}}_{\mathrm{CNL}}$$

3 $$\mathrm{MCC}=\frac{\mathrm{TP}\cdot \mathrm{TN}-\mathrm{FP}\cdot \mathrm{FN}}{\sqrt{\left(\mathrm{TP}+\mathrm{FP}\right)\left(\mathrm{TP}+\mathrm{FN}\right)\left(\mathrm{TN}+\mathrm{FP}\right)\left(\mathrm{TN}+\mathrm{FN}\right)}}$$

4 $$F1=\frac{\mathrm{TP}}{\mathrm{TP}+\frac{1}{2}\left(\mathrm{FP}+\mathrm{FN}\right)}$$

5 $$\mathrm{re}call=\frac{\mathrm{TP}}{\mathrm{TP}+\mathrm{FN}}$$

6 $$h_{\mathrm{ii}}=x_{i}^{T}{\left(X^{T}X\right)}^{-1}x_{i}$$

7 $$P\left(\mathrm{id}entificationforeachRPLC/HRMStensor\right)=\frac{1}{N}$$

8 $$P\left(identification\;for\;each\;RPLC/HRMS\;tensorin\;TopX\;of\;\mathrm{hi}ts\right)=\frac{N}{X}$$

## Supplementary Material



## Data Availability

Three preprocessed experimental data sets are provided for demonstration at https://github.com/TommyNHL/exposomeIDProba/tree/main/demo_data (CSV). List of compounds used for modeling (**files 1 and 3**), their predicted RTI values (**file 4**), the selected MOIs (**file 2**), and the spectral matching data for Pencycuron (**file 5**) are available at 10.5281/zenodo.16402775 (XLSX).

## Notes

Experimental methods for ULSA, semisynthetic and experimental data preparation, and the identification workflow; code availability for model development and application; technical details presented by a roadmap (Figure S01); scatter plots of correlation between *RTI*_*MF*_ and *RTI*_*CNL*_ values (Figure S02); line plots representing the performance of Model 3 (Figure S03); heatmap representing the IPs by ranking (Figure S04); histograms showing the distribution of *TP* and *TN* spectral matches (Figures S05–S13); and plots indicating the performance of Model 3 during feature and model selection (Figures S14–S16) (PDF)
