# Supplementary material for: Machine Learning for Enhanced Identification Probability in RPLC/HRMS Nontargeted Workflows
Source: Anal Chem. 2025 Aug 12;97(33):18028–35. doi: 10.1021/acs.analchem.5c01873 (PMC12392258; doi:10.1021/acs.analchem.5c01873)
Supplement: Supplementary file 1 [file ac5c01873_si_001.pdf]

Supporting Information for

Machine Learning for Enhanced Identification Probability in RPLC/HRMS Non-Targeted Workflows

Hiu-Lok Ngan,† Viktoriia Turkina,§ Denice van Herwerden,§ Hong Yan,†,‡ Zongwei Cai,*,† and Saer Samanipour*,§,∥

†State Key Laboratory of Environmental and Biological Analysis, Department of Chemistry, Hong Kong Baptist University, Kowloon, Hong Kong 999077, P. R. China
‡Department of Biology, Hong Kong Baptist University, Kowloon, Hong Kong 999077 P. R. China
§Van 't Hoff Institute for Molecular Sciences (HIMS), University of Amsterdam, Amsterdam 1098 XH, The Netherlands
∥UvA Data Science Center, University of Amsterdam, Amsterdam 1012 WP, The Netherlands

***Corresponding authors: Drs. Zongwei Cai and Saer Samanipour**
E-mails: zwcai@hkbu.edu.hk; s.samanipour@uva.nl

TABLE OF CONTENTS

ADDITIONAL TEXT S-2
Universal Library Search Algorithm (ULSA) Parameters S-2
Semisynthetic Data Preparation S-2
RPLC/HRMS for Pesticides' Analyses S-2
NTA Data Pre-Processing by jHRMS ToolBox S-3
Technical Details of This Work S-3
Code Availability for Model Development and Application S-10
Supporting Formulae and Equations S-11
References for the Additional Text S-12
ADDITIONAL FIGURES S-13
F igure S01: Technical Details Presented by A Roadmap S-13
Figure S02: Correlation Between the Predicted Values by 2 Different RTI Systems S-15
Figure S03: Performance of the Optimal Model 3 ..... S-16
Figure S04: Identification Probability Assessment by Ranking. S-17
Figure S05–S13: Distribution of True Positive and True Negative Spectral Matches S-19
Figure S14–S16: Performance of Model 3 During Feature and Model Selection S-28
ADDITIONAL TABLES S-31
Table S1: Computational Power and Packages Used for Modeling. S-31
Table S2: NTA Data Pre-Processing by jHRMS ToolBox. S-32
Table S3: Hyper-parameters Optimization History for Model 2. ..... S-34
Table S4: Hyper-parameters Optimization History for Model 3. ..... S-36

ADDITIONAL TEXT

Universal Library Search Algorithm (ULSA) Parameters

ULSA final scores are ranged from 0 to 7, with higher values indicating increased reliability of the individual spectral match. The feature "FinalScoreRatio" (S_8) was calculated by dividing the final score by 7. In **eq S1**, MS_{tol} represents the user-defined *m/z* tolerance in Daltons for an ion to be accepted as a positive match. The 7 features (S_{1-7}) that were extracted from the spectra for feature selection in Model 3 included: S_{1-2}, the fractions of matched fragments to the reference spectra (denoted as $f_{matched}/f_{reference}$ in **eq S1**) and to the total fragments in the user spectrum ($f_{matched}/f_{user}$); S_3, the observed mass error in the precursor ion ($MS_{err,pre}$); S_4, the absolute average mass error of all matched fragment ions ($|MS_{err,frag}|$); S_5, the standard deviation of mass error for matched fragments ($STD_{err,frag}$); and S_{6-7}, the forward ($MF_{forward}$) and reverse ($MF_{reverse}$) match factors calculated via dot product.

Semisynthetic Data Preparation

HRMS spectra with a mass resolution > 5,000, acquired using an ESI+ source, were randomly selected from databases such as MassBank EU, MoNA, GNPS, and NIST. To mimic false negative (*FN*) ions (i.e., loss ions) caused by ion suppression or acquisition defects, 10% fragments were removed randomly. Data augmentation was applied by adding noise to simulate false positive (*FP*) ions. For instance, if the mass tolerance for MS/MS spectral matching was set to ±20 mDa, *FP* ions with random mass errors in a range of ±30 mDa were incorporated to the accurate masses of each fragment ion.

RPLC/HRMS for Pesticides' Analyses

After developing all the predictive models, they were integrated into the non-targeted analysis (NTA) pipeline for aiding pesticide annotation as a real-world application in drinking water and tea matrix. A set of RPLC-MS/MS data from pesticides-spiked black tea extract

samples, as previously reported in a cumulative neutral loss (CNL) model development study.[1] The pesticide standards were part of the LC/MS Pesticide Comprehensive Mix Kit (Part Number 5190-0551) from Agilent Technologies. Pesticide mixtures were spiked in solvent blank and 10× and 100× diluted black tea matrix respectively, resulting 1, 2.5, 5, 10, 25, 50, 100, and 1,000 ppb final solutions. The details of data acquisition were outlined in the prior CNL model study.[1] The optimal binary classification model was tested on an external independent dataset, which comprised of experimental data of pesticides spiked into blank samples at varying concentrations (1, 2.5, 5, 10, 25, 50, 100, and 1,000 ppb).

NTA Data Pre-Processing by jHRMS ToolBox

To extract HRMS information required as the model input, a user interface, namely "jHRMS ToolBox", was previously developed for no-code application.[2] Detailed descriptions of its component modules and their modules' input/output were summarized in **Table S2** for facilitating MS community to reassemble the pipeline. To introduce, firstly, the upstream processing of LC/HRMS files requiring the open MS format, ".mzXML". Raw data files conversion was accomplished using the "MS_Import" module, built on MSConvertGUI (64-bit, ProteoWizard[3]). Secondly, this ecosystem also incorporates the self-adjusting feature detection (SAFD) algorithm for extracting LC-MS/MS features.[4] Thirdly, componentization was achieved by executing the "CompCreate" module, which correlates the extracted features with the probabilistic CNL model.[1] Finally, the resultant MS/MS spectra were then matched using ULSA.[5] It returned a spreadsheet that contains necessary information for the identification workflow, including the suggested compound, *m/z* value of the molecular ion, a list of *m/z* values of the correlated fragments, and the scores S_{1-8}.

Technical Details of This Work

The roadmap for this work with detailed information is illustrated in **Figure S01**. In this work, 3 supervised learning models were newly developed. The following additional text

covers all technical details of machine learning (ML) model training materials, data pre-processing, model hyperparameters optimization, model and feature selection, model training, validating, and testing.

Technical Details of Model 1 Development. Development of the first model (Model 1) was similar to a previous work with some modifications.[6] As shown in **Figure S01A**, Model 1 is a random forest (RF) regression (reg.) model that correlates molecular fingerprint (MF) to the true retention time index (RTI) values of calibrants from 3 comparable scales. It is used for predicting the expected RTI on a harmonized scale by analyzing MFs of a chemical compound with simplified molecular-input line-entry system (SMILES) identity.

Date Pre-Processing of Model 1's Training Materials. The training materials of Model 1 are consisted of 6,311 queries of compound calibrants' true RTI from 3 comparable RTI scales: the C3–14 n-alkylamide system (Amide),[7] the system developed by Aalizadeh et al. from the University of Athens (UoA),[8] and the C0–23 cocamide diethanolamine homologous series (Cocamide).[9] There are 6,311 true RTI values in total for 5,950 distinct calibrants with SMILES identities (IDs). The list of compounds' SMILES IDs involved was attached as additional spreadsheet file (see **file 1** at 10.5281/zenodo.16402775). As similar to the previous work,[6] this model correlates 2 systems of 2D molecular descriptors to RTIs. The first system, AtomPairs2DFingerprintCount (APC2D), characterizes molecules using 780 bits that count the frequency of atom appearances within specified distances between atom pairs. The second system utilizes the 881-bit PubChem Fingerprinter (PubChem). In Model 1's dataframe (df), MFs were computed using PaDEL for each canonical SMILES-identified molecule.[10] From the 881 MF features in the PubChem system, only 148 bits (i.e., from the 115th to the 262nd) were selected and condensed to 10 features. As a result, each compound was represented by a 790-bit MF features.

Model 1 Training, Validating, and Testing. To perform train/test split on the prepared materials, stratification was performed using 5 classes based on the leverage values calculated from the independent variable MFs. An 8:2 train/test split ratio was selected for stratified data splitting. Transferable prediction of RTI values was achieved by training a model on 4,713 calibrants with 5,048 true RTIs across 3 comparable RTI scales in RPLC. Model performance was assessed by accuracy of the predicted RTI value on the harmonized scale was tested by 1,263 true values of 1,237 untrained calibrants in terms of mean absolute error. The RF reg. model was trained by 200 trees, with a minimum of tree leaves of 8 and a maximum of one-third of the features for each tree's training.

Applicability Domain (AD) of Model 1. A new compound's inclusion within the AD of this model is determined by the 95% leverage threshold (0.275) based on the 80% split MF dataset. If a leverage value of a compound exceeded 0.275, the predicted molecular RTI might have high uncertainty, indicating that Model 1 may not effectively predict the expected RTI value for this compound that is outside the prerequired chemical space.

Technical Details of Model 2 Development. Development of the second model (Model 2) was similar to a previous work with some modifications.[11] As shown in **Figure S01B**, Model 2 is another RF reg. model that correlates neutral loss masses to the expected RTIs that were predicted from Model 1. Model 2 is used for predicting the expected RTI on a harmonized scale by using the extracted tandem mass spectrometry (MS/MS) spectral information of a chemical compound.

Date Pre-Processing of Model 2's Training Materials. Development of Model 2 initially utilized 1,095,389 experimental MS/MS spectra from various external spectral databases (dbs), including MassBank EU, MoNA, and NIST LC-MS/MS. As only empirical positive electrospray ionization (ESI+) MS/MS were considered, 277,976 queries were excluded. Neutral loss (NL) was calculated by subtracting the mass-to-charge ratio (*m/z*) values

of molecular ion by that of fragment ions. There were 27,434,522 precise measures in total, and binning to a precision of ±0.005 Da resulted in 15,994 distinct NL masses. Given that using a bin size of 0.01 Da over the *m/z* range of 50 to 1,000 results in approximately 95,000 bins which is computationally expensive, a list of 16,022 high probability masses-of-interest (MOIs) pre-selected based on a previous study on cumulative NL (CNL).[1] The list is available in the additional spreadsheet file (see **file 2** at 10.5281/zenodo.16402775). In Model 2's df, 123,728 query spectra were removed as none of the CNL mass was in the list of MOIs, remaining 693,685 spectra with 15,961 MOIs that were binned from 25,497,040 NLs. Each query MS/MS spectrum contained at least 2 MOIs. Their presences were encoded in a one-hot encoding fashion, assigning a value of 0 or 1 to each sample entry if a MOI was absent or present, respectively. Moreover, a float vector of monoisotopic masses was included as the first column, with values scaled to lie between 0 and 1. This feature was previously reported to be one of the most discriminative to CNL-based model due to its intrinsic relationship with molecular weight.[11]

Parallelly, in the available MS/MS spectral dbs, 31,402 compounds were InChIKey IDs registered. Among them, 30,684 compounds were also identified by SMILES IDs, and thus could be proceeded for MF feature engineering and MF-to-RTI prediction. Within the 693,685 spectra, 4 spectra were excluded because the prediction of their compounds' MF-derived RTIs were out of the AD of Model 1. Eventually, there were 693,681 remaining spectra, corresponding to 27,211 distinct compounds that were identified by both InChIKey and SMILES IDs, being assigned the expected RTIs that were predicted by Model 1.

Model 2 Training, Validating, and Testing. To perform train/test split on the prepared materials, stratification was performed using 5 classes based on the leverage values calculated from the independent variable MFs. In the MF df, 663,001 queries were added based on the frequency of appearance in the CNL df, resulting a df with same number of rows as the CNL

df. The frequency of appearance of spectra that were identified by compounds' InChIKey IDs were listed in the additional spreadsheet file, see **file 3** at 10.5281/zenodo.16402775). Due to the computational limitations, a 7:3 train/test split ratio was selected for stratified splitting. Transferable prediction of RTI values was achieved by training a model on 485,577 spectra with the expected RTIs on a harmonized scale. To assess model transferability, 208,104 CNL-derived RTI values of the 30% split testing data in Model 2 was tested by the predicted RTI values from Model 1.

For hyper-parameters selection, the 70% split training dataset was further divided into a 9:1 split for internal training and validation, with optimization monitored by the coefficient of correlation (R^2) value. Three-fold cross-validation (CV) was chosen over 10-fold CV due to similar performance and reduced computational time. Detailed hyperparameter tuning history can be found in **Table S3**. The RF model was trained by 350 trees, with a minimum of tree leaves of 2 and a maximum of one-third of the features for each tree's training.

Applicability Domain (AD) of Model 2. A new compound's inclusion within the AD of Model 2 is determined by the 95% leverage threshold (0.146) based on the 70% split CNL dataset. If a leverage value of a compound exceeded 0.146, the predicted molecular RTI might have high uncertainty, indicating that Model 2 may not effectively predict the expected RTI value for this compound that is outside the prerequired chemical space.

Technical Details of Model 3 Development. As shown in **Figure S01C**, Model 3 is a binary classification (class.) model that integrates features derived from both RTI and *m/z* domains. It aimed at determining whether a reference spectrum matched by universal MS/MS spectral library search should be accepted or rejected, given that its spectral matching score is higher than the half of total score.

Data Pre-Processing of Model 3's Training Materials. The development of Model 3 employed semi-synthetic HRMS data. A total of 4,368,902 query spectra were prepared and

then proceeded to universal library search, which can be implemented by ULSA,[5] obtaining 8 spectral match-score parameters, namely "RefMatchFragRatio" (S_1), "UsrMatchFragRatio" (S_2), "MS1Error" (S_3) in 2-way. "MS2Error" (S_4) in 1-way, "MS2ErrorStd" (S_5), "DirectMatch" (S_6), "ReverseMatch" (S_7), and "FinalScoreRatio" (S_8). MF-derived RTIs were computed by Model 1 deployment for the library search-suggested compounds. The MF-derived RTIs were library search's measured values. There were 96,114 queries excluded because the corresponding compounds of the matched spectra were lack of SMILES IDs for obtaining MF features. Parallelly, CNL-derived RTIs were computed as actual values. Further 32,262 queries were removed during CNL df preparation. Since all matched spectra with a "FinalScoreRatio" (S_8) < 0.50 are in 100% of *TPs* within the remaining 4,240,526 query spectral match results and the independent experimental pesticides-spiked samples' data, only the data with $S_8 < 0.50$ were determined for training (and model application). Ultimately, 2,107,700 spectra were proceeded to strategic train/test split.

To perform train/test split on the prepared materials, stratification was performed using 5 classes based on the leverage values calculated from the independent variable monoisotopic mass and 15,961 CNL masses. An 8:2 train/test split ratio was selected for stratified splitting. *TP*/*TN* reference spectral match binary classification for molecular features with the spectral matching scores higher than the half of total score was achieved by training and testing a model on 1,686,319 and 421,381 spectral matching results, respectively. Among the training samples, 1,535,009 spectra were *TN* and labeled as "0", while 151,310 instances represented *TP* (labeled as "1").

Feature Engineering and Selection for Model 3. On top of the 8 scores returning from library search are considered as feature candidates of Model 3, a 2-way RTI difference, namely "DeltaRi", was created by subtracting measured RTI (MF-derived RTI) by actual RTI (CNL-derived RTI). Additionally, the feature "MONOISOTOPICMASS/1000" was incorporated.

Exploratory data analysis (EDA) was conducted by assessing distribution of individual feature candidate in *TP* and *TN* data. Histograms revealed that features such as “RefMatchFragRatio” (**Figures S05A**, **D**, **G**, and **J**), “UsrMatchFragRatio” (**Figures S06A**, **D**, **G**, and **J**), “FinalScoreRatio” (**Figures S07A**, **D**, **G**, and **J**), “MONOISOTOPICMASS/1000” (**Figures S08A**, **D**, **G**, and **J**) exhibited right-skewed distributions. Log-transformation was applied to achieve relatively normal distribution (subplots B, E, H, and K of **Figures S05–S08**). For feature “MS1Error”, its data distribution was similar to that of “DeltaRi”. The 2-sided data (subplots B, E, H, and K of **Figures S09–S10**) showed better normality compared to absolute data (subplots A, D, G, and J of **Figures S09–S10**). In contrast, for the 1-sided data of “MS2Error” and “MS2ErrorStd”, although they were right-skewed nature (subplots A, C, E, and G of **Figures S11–S12**), log-transformation was forbidden as most counts were 0. For the features “DirectMatch” and “ReverseMatch”, original values (subplots A–B, E–F, I–J, and M–N of **Figure S13**) showed left-skewed distribution. The difference between “DirectMatch” and “ReverseMatch” was computed to create a new feature, namely “MatchDiff”, which provided a better distribution (subplots C, G, K, and O of **Figure S13**). All data were undergone mean normalization prior to feature selection and model performance comparisons, essential for the linear models, although unnecessary for tree-based models.

Feature exclusion was assessed by Pearson correlation to eliminate redundant features. As shown in **Figure S14**, the feature “MS2Error” exhibited a high correlation (> 0.8) with both features “MS2ErrorStd” and “RefMatchFragRatio” across all datasets, while no strong correlation between features “MS2ErrorStd” and “RefMatchFragRatio”. Hence, feature “MS2Error” was excluded from the panel. The discriminative features include logarithm of S_1, S_3, S_5, logarithm of S_8, logarithm of a value of monoisotopic mass/1000, and “DeltaRi”.

Model Selection and Hyperparameter Tuning for Model 3. To fairly compare different ML algorithms, 9 additional replicates of *TP* spectra were added used to balance the training

dataset. The performances of various ML algorithms were evaluated on both internal 3-fold CV datasets and an independent dataset, which was a set of pre-processed experimental data of pesticides blank spikes at varying concentrations (1, 2.5, 5, 10, 25, 50, 100, and 1,000 ppb). A series of hyperparameter tuning across various classification models, including logistic regression (LR), decision tree (DT), RF, and k-Nearest neighbors (KNN). During model selection, further feature selection was performed by prioritizing those features with the most negative contribution, following by model sensitivity analyses of the excluded feature(s). Detailed hyperparameter tuning history for all models can be found in **Table S4**. Hyperparameters were primarily optimized based on the weighted $F1$ score. For real samples with presence of matrix, recall was examined as negatives were unknown.

As indicated in **Figure S15**, RF and KNN models emerged as superior performers compared to DT and LR models in both training and testing datasets. The KNN models, in particular, demonstrated slightly better performance for the pesticides-spiked blank (validation dataset in **Figure S16A**) and for the tea solution mixture (real sample dataset in **Figure S16B**), prompting its use for further analysis. Permutation feature importance was applied with 10 replicates to identify and exclude the least impactful features. As the logarithm of feature “UsrMatchFragRatio”, namely “log(UsrMatchFragRatio)”, had the lowest importance to the model, its removal improved the predictive power of all models.

Model 3 Training. The optimal model (Model 3) is a 6-component KNN model that incorporated features $\log(S_1)$, S_3, S_5, $\log(S_8)$, log(MONOISOTOPICMASS/1000), and RTI error (“DeltaRi”). It was trained by 379 neighbors, with a leaf size of 300 and a power parameter of 2 (i.e., Euclidean distance, L2) for the Minkowski metric.

Code Availability for Model Development and Application

All scripts for ML modeling and data visualization were written in Julia v.1.6 using Visual Studio Code (Microsoft), while some data visualization was conducted in Python v.3.10 on

Jupyter Notebook (Anaconda3). The scripts for models' development can be found at https://github.com/TommyNHL/exposomeProjUvA. In addition, the scripts for models' applications and the 3 developed models are available in the following repositories respectively: https://github.com/TommyNHL/exposomeIDProba (scripts); and https://bitbucket.org/hiulokngan/modelsExposomeProjUvA (models). Details regarding computational power and packages used can be found in **Table S1**. Any updates will be published on both repositories with a citation of the final DOI for this work.

Supporting Formulae and Equations

**Eq S1**	$S_8 = \frac{f_{matched}}{f_{user}} + \frac{f_{matched}}{f_{reference}} + \frac{MS_{tol} - MS_{err,pre}}{MS_{tol}} + \frac{f_{matched}}{f_{reference}} \cdot \frac{MS_{tol} - \lvert MS_{err,frag} \rvert}{MS_{tol}} + \frac{f_{matched}}{f_{reference}} \cdot \frac{2 \cdot MS_{tol} - STD_{err,frag}}{2 \cdot MS_{tol}} + MF_{forward} + MF_{reverse} = S_1 + S_2 + \frac{MS_{tol} - S_3}{MS_{tol}} + S_2 \cdot \frac{MS_{tol} - S_4}{MS_{tol}} + S_2 \cdot \frac{2 \cdot MS_{tol} - S_5}{2 \cdot MS_{tol}} + S_6 + S_7$

References for the Additional Text

(1) Van Herwerden, D.; O'Brien, J. W.; Lege, S.; Pirok, B. W. J.; Thomas, K. V.; Samanipour, S. Cumulative Neutral Loss Model for Fragment Deconvolution in Electrospray Ionization High-Resolution Mass Spectrometry Data. *Anal. Chem.* **2023**, *95* (33), 12247–12255.

(2) Van Herwerden, D.; Kant, E.; Jackson, M.; Fender, C.; Garcia-Jaramillo, M.; O'Brien, J.; Thomas, K.; Samanipour, S. Modular Open-Access and Open-Source Julia Language Toolbox for Processing of HRMS Data: JHRMSToolBox. *ChemRxiv* **2024**, DOI:10.26434/chemrxiv-2024-b713v-v2.

(3) Chambers, M. C.; MacLean, B.; Burke, R.; Amodei, D.; Ruderman, D. L.; Neumann, S.; Gatto, L.; Fischer, B.; Pratt, B.; Egertson, J.; Hoff, K.; Kessner, D.; Tasman, N.; Shulman, N.; Frewen, B.; Baker, T. A.; Brusniak, M. Y.; Paulse, C.; Creasy, D.; Flashner, L.; Kani, K.; Moulding, C.; Seymour, S. L.; Nuwaysir, L. M.; Lefebvre, B.; Kuhlmann, F.; Roark, J.; Rainer, P.; Detlev, S.; Hemenway, T.; Huhmer, A.; Langridge, J.; Connolly, B.; Chadick, T.; Holly, K.; Eckels, J.; Deutsch, E. W.; Moritz, R. L.; Katz, J. E.; Agus, D. B.; MacCoss, M.; Tabb, D. L.; Mallick, P. A Cross-Platform Toolkit for Mass Spectrometry and Proteomics. *Nat. Biotechnol.* **2012**, *30* (10), 918–920.

(4) Samanipour, S.; O'Brien, J. W.; Reid, M. J.; Thomas, K. V. Self Adjusting Algorithm for the Nontargeted Feature Detection of High Resolution Mass Spectrometry Coupled with Liquid Chromatography Profile Data. *Anal. Chem.* **2019**, *91* (16), 10800–10807.

(5) Samanipour, S.; Reid, M. J.; Bæk, K.; Thomas, K. V. Combining a Deconvolution and a Universal Library Search Algorithm for the Nontarget Analysis of Data-Independent Acquisition Mode Liquid Chromatography-High-Resolution Mass Spectrometry Results. *Environ. Sci. Technol.* **2018**, *52* (8), 4694–4701.

(6) Van Herwerden, D.; Nikolopoulos, A.; Barron, L. P.; O'Brien, J. W.; Pirok, B. W. J.; Thomas, K. V.; Samanipour, S. Exploring the Chemical Subspace of RPLC: A Data Driven Approach. *Anal. Chim. Acta* **2024**, *1317*, No. 342869.

(7) Hall, L. M.; Hill, D. W.; Menikarachchi, L. C.; Chen, M. H.; Hall, L. H.; Grant, D. F. Optimizing Artificial Neural Network Models for Metabolomics and Systems Biology: An Example Using HPLC Retention Index Data. *Bioanalysis* **2015**, *7* (8), 939–955.

(8) Aalizadeh, R.; Alygizakis, N. A.; Schymanski, E. L.; Krauss, M.; Schulze, T.; Ibáñez, M.; McEachran, A. D.; Chao, A.; Williams, A. J.; Gago-Ferrero, P.; Covaci, A.; Moschet, C.; Young, T. M.; Hollender, J.; Slobodnik, J.; Thomaidis, N. S. Development and Application of Liquid Chromatographic Retention Time Indices in HRMS-Based Suspect and Nontarget Screening. *Anal. Chem.* **2021**, *93* (33), 11601–11611.

(9) Aalizadeh, R.; Nikolopoulou, V.; Thomaidis, N. S. Development of Liquid Chromatographic Retention Index Based on Cocamide Diethanolamine Homologous Series (C(n)-DEA). *Anal. Chem.* **2022**, *94* (46), 15987–15996.

(10) Yap, C. W. PaDEL-Descriptor: An Open Source Software to Calculate Molecular Descriptors and Fingerprints. *J. Comput. Chem.* **2011**, *32* (7), 1466–1474.

(11) Boelrijk, J.; van Herwerden, D.; Ensing, B.; Forré, P.; Samanipour, S. Predicting RP-LC Retention Indices of Structurally Unknown Chemicals from Mass Spectrometry Data. *J. Cheminf.* **2023**, *15*, No. 28.

ADDITIONAL FIGURES

Figure S01

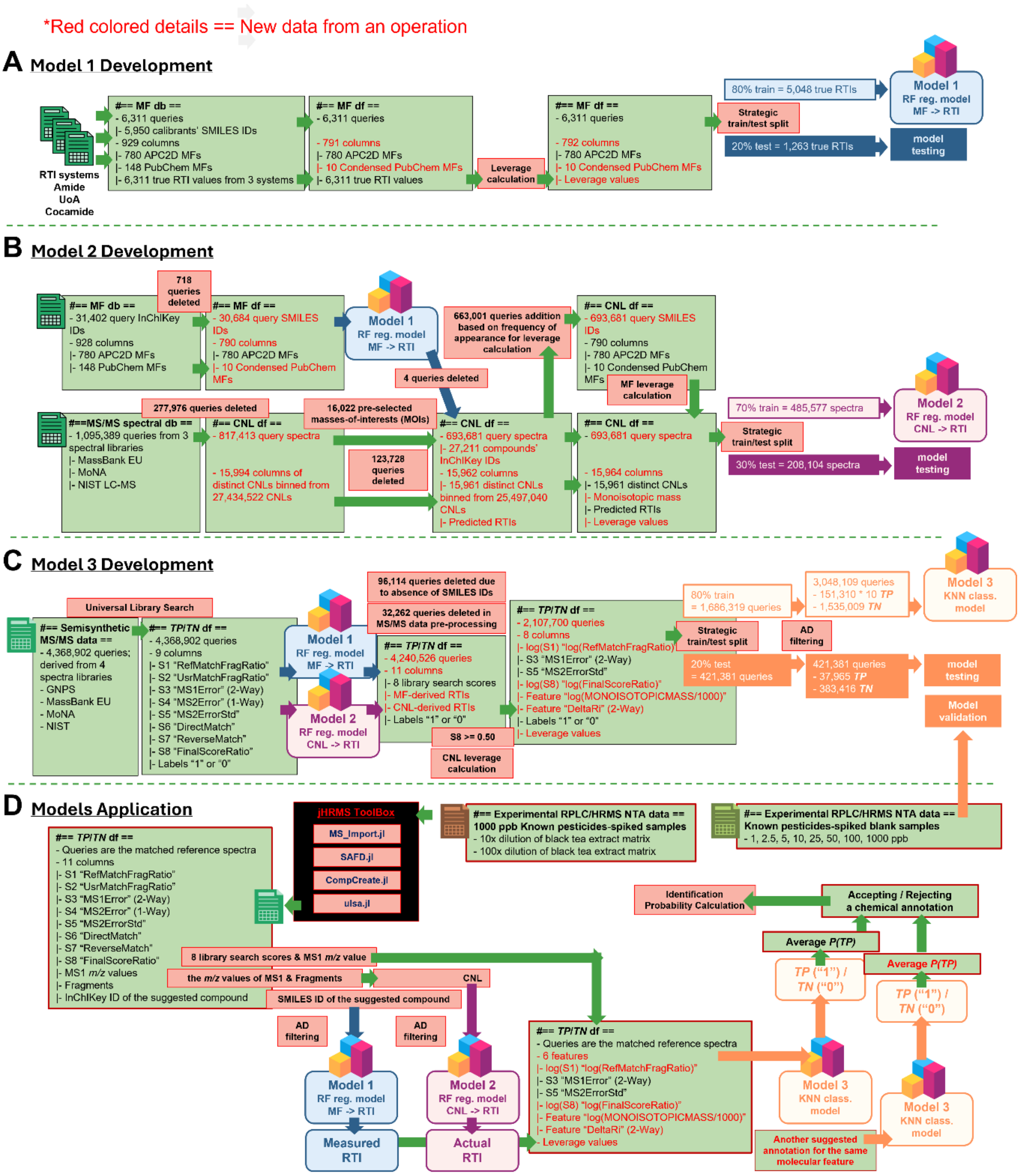

Figure S01: A diagram of roadmap in this study with detailed information. Workflows of developing **(A)** Model 1, **(B)** Model 2, and **(C)** Model 3. **(D)** The identification workflow in this work. *Abbreviations: MF, Molecular fingerprint; db, Database; SMILES, Simplified molecular-input line-entry system; APC2D,*

AtomPairs2DFingerprintCount; PubChem, PubChem Fingerprinter; df, dataframe; RF, Random forest; RTI, Retention time index; reg., Regression; MoNA, MassBank of North America; CNL, cumulative neutral loss; ULSA, Universal Library Search Algorithm; TP, True positive; TN, True negative; KNN, k-Nearest neighbors; class., Classification; RPLC, Reversed-phase lipid chromatography.

Figure S02

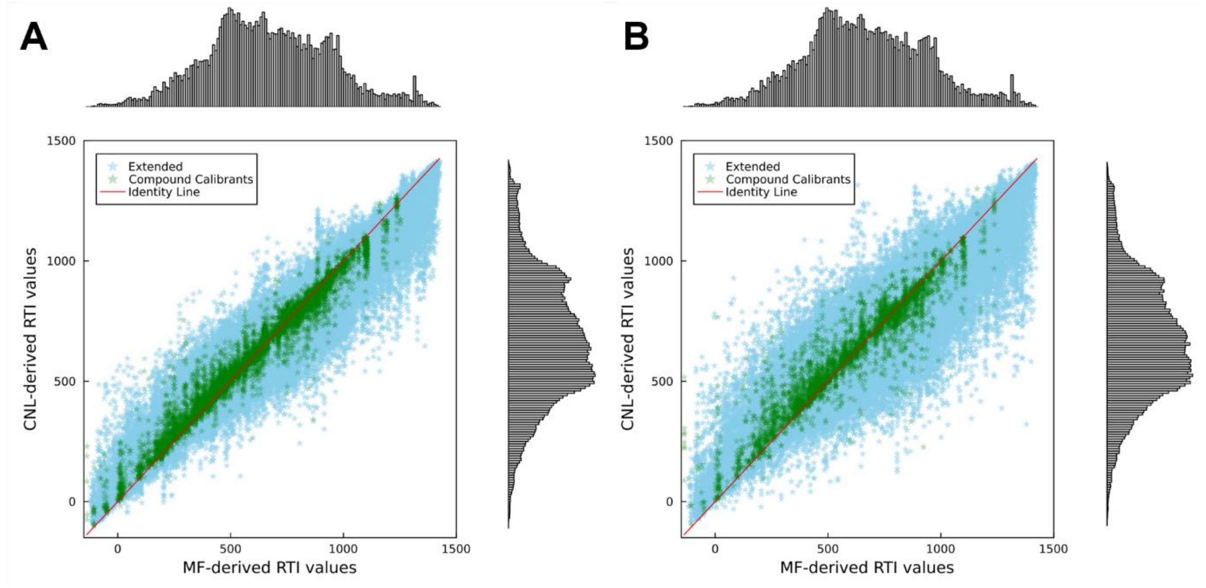

Figure S02: Correlation plots of molecular fingerprint (MF)-derived and cumulative neutral loss (CNL)-derived RTIs. Correlation plots showing correspondence between molecular fingerprint (MF)-derived and cumulative neutral loss (CNL)-derived RTIs for **(A)** training and **(B)** testing datasets of Model 2. Green dots representing compounds used in Model 1's training, yet sky-blue dots indicating new compounds within the RPLC chemical space after applicability domain filtering.

Figure S03

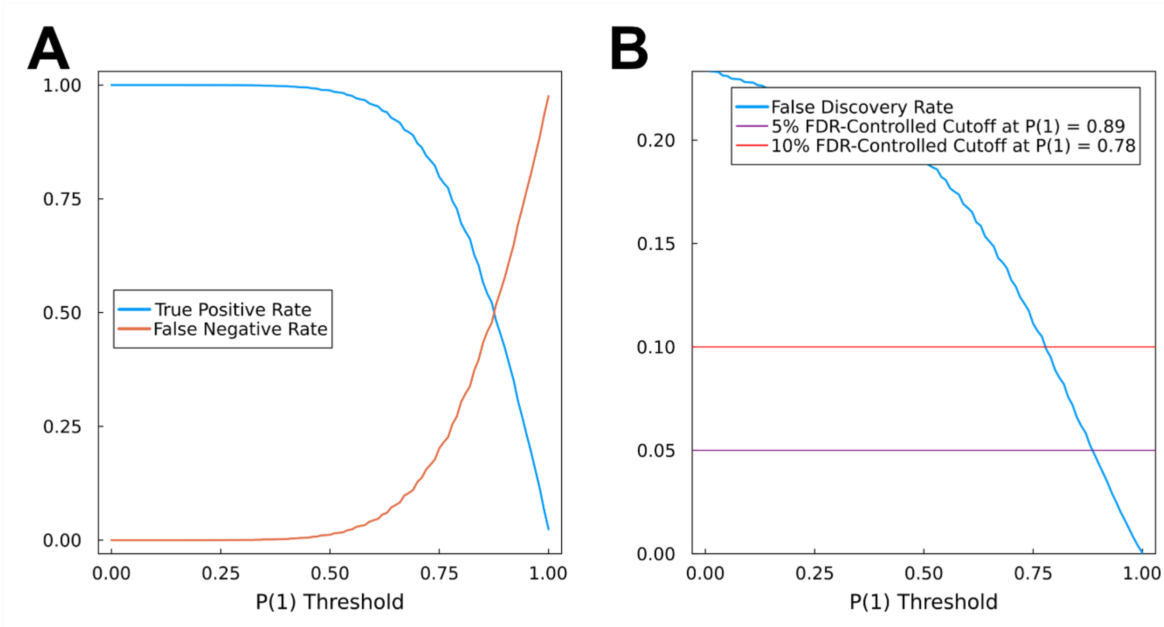

Figure S03: Performance assessment of Model 3. (A) True positive (*TP*) and false negative rates and **(B)** false discovery rates (FDR) across various probability of true positive (*P*(1) or *P*(*TP*)) thresholds.

Figure S04

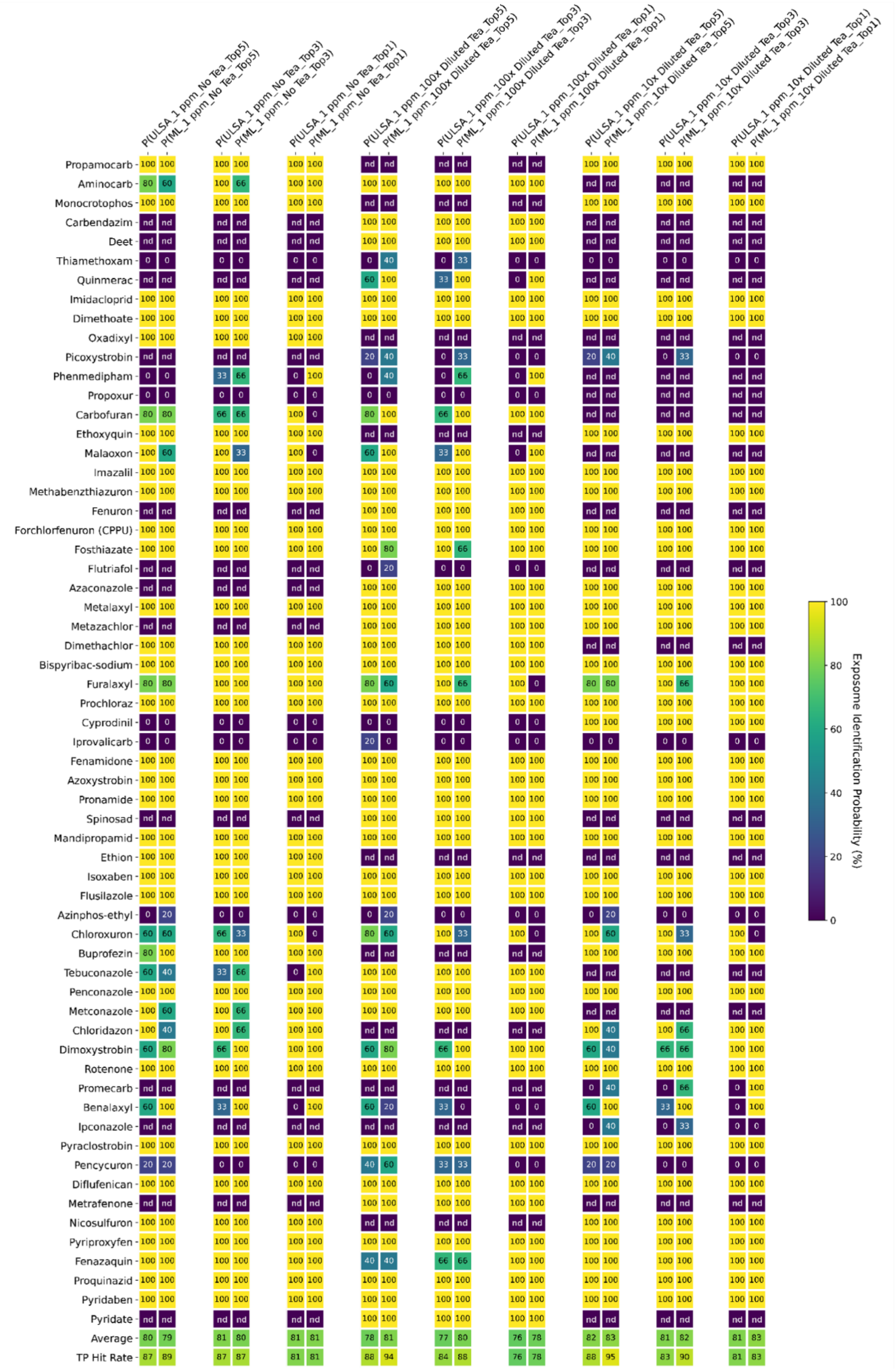

Figure S04: Identification probability assessment by ranking. Individual matches were ranked by matching score “FinalScoreRatio” for Universal Library Search Algorithm

(ULSA) and by individual *P*(*TP*) value for machine learning (ML) integration. ML incorporation with ULSA slightly enhanced average IP by 1–3% in top-5, top-3, and top-1 hits, showing structure assignment by ranking may mislead the result. True positive (*TP*) hit rates were calculated as the ratio of molecular annotations with any *TP* hit at least once in the top ranks to the total identified spectra. *Abbreviations: nd, not detected.*

Figure S05

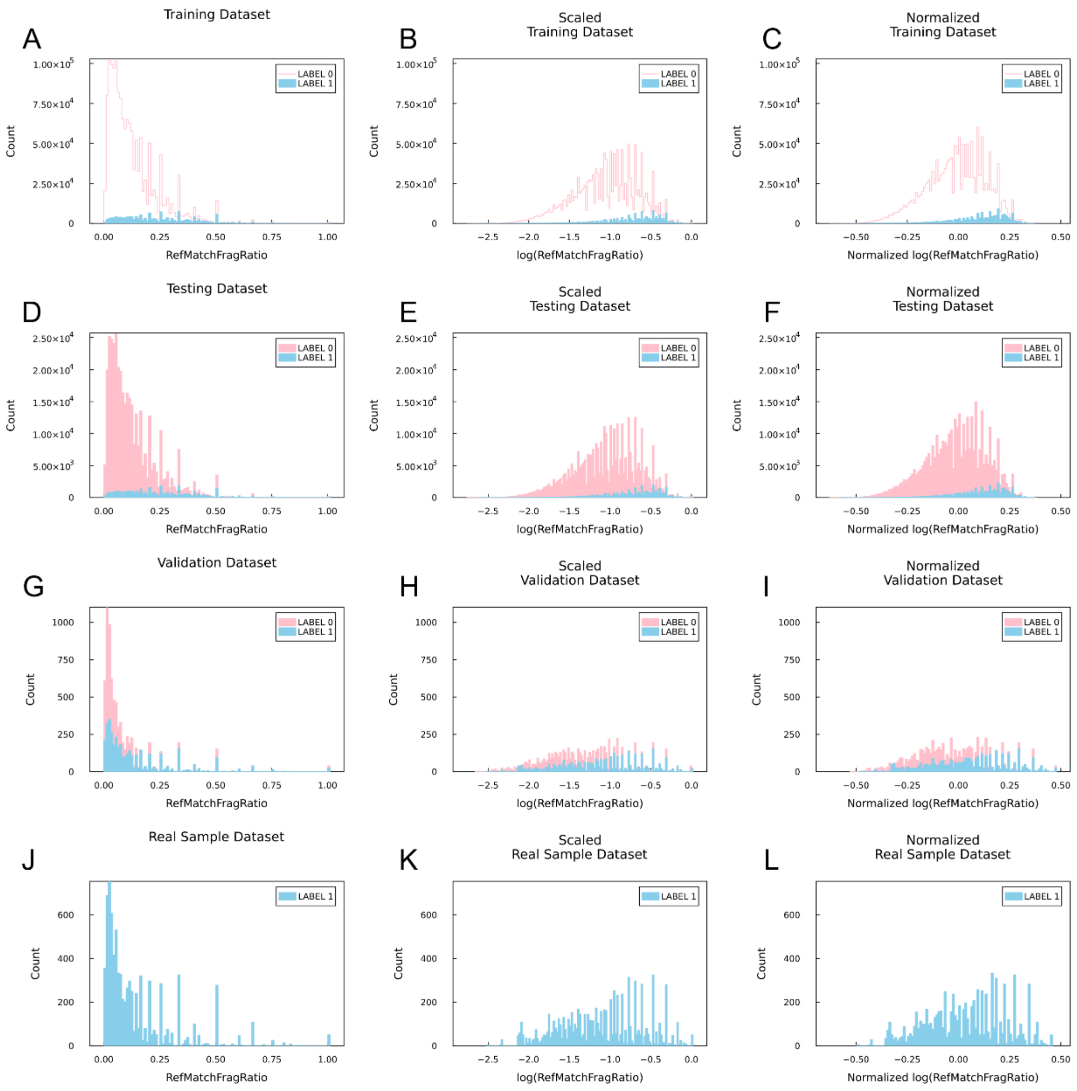

Figure S05: Distribution of the feature "RefMatchFragRatio" (S1) in true positives and true negatives data. Histograms illustrating distributions of true positive (LABEL 1) and true negative (LABEL 0) samples for **(A–C)** training, **(D–F)** testing, **(G–I)** validation by pesticides blank spike, and **(J–L)** real sample analyses for the pesticides-spiked black tea sample datasets. Original values (subplots A, D, G, and J) showed right-skewed distributions. Log-transformation (subplots B, E, and H) and normalization by mean (subplots C, F, and I) showed the distributions of data with labels "1" and "0" were slightly split.

Figure S06

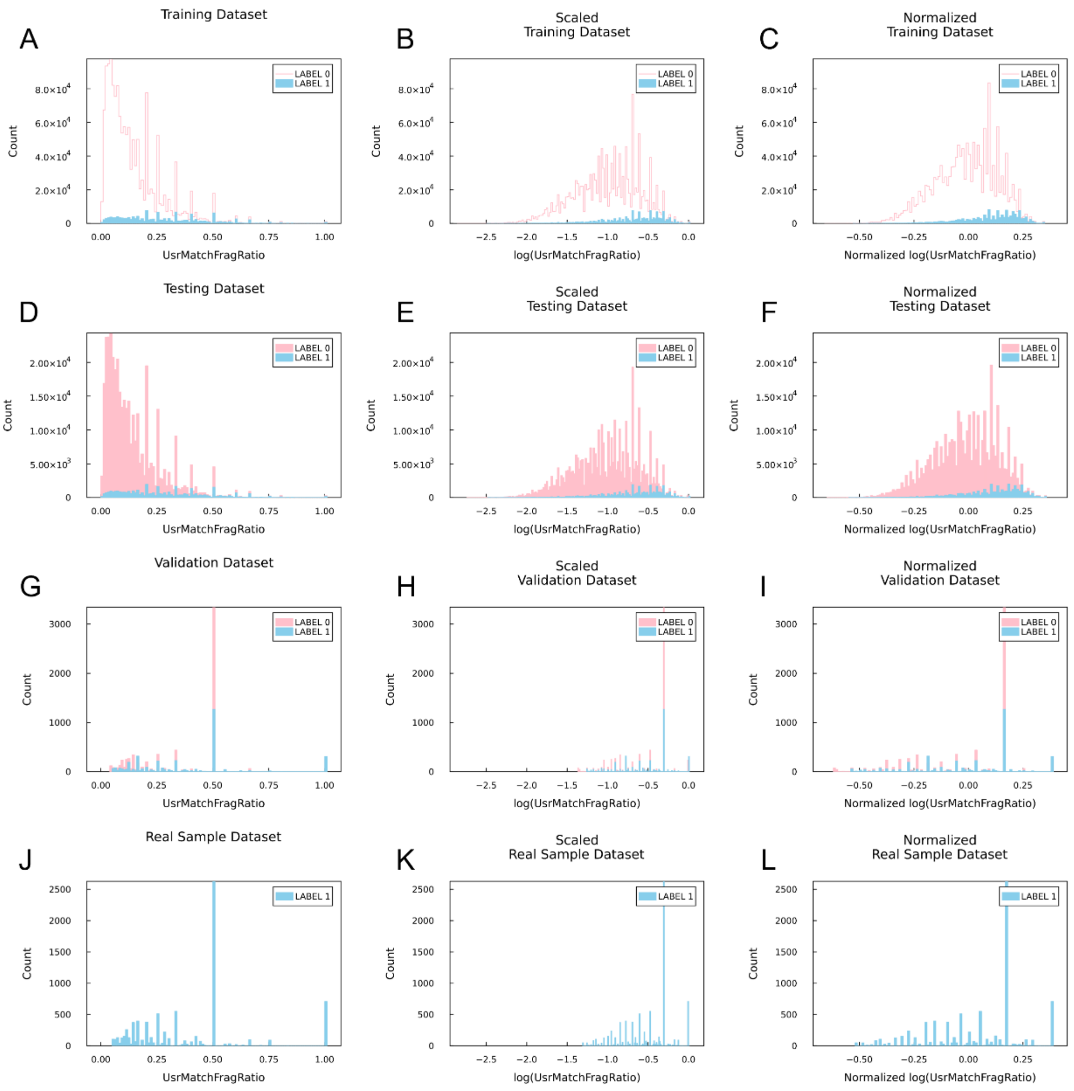

Figure S06: Distribution of the feature "UsrMatchFragRatio" (S2) in true positives and true negatives data. Histograms illustrating distributions of true positive (LABEL 1) and true negative (LABEL 0) samples for **(A–C)** training, **(D–F)** testing, **(G–I)** validation by pesticides blank spike, and **(J–L)** real sample analyses for the pesticides-spiked black tea sample datasets. Original values (subplots A, D, G, and J) showed right-skewed distributions. Log-transformation (subplots B, E, and H) and normalization by mean (subplots C, F, and I) showed the distributions of data with labels "1" and "0" were slightly split.

Figure S07

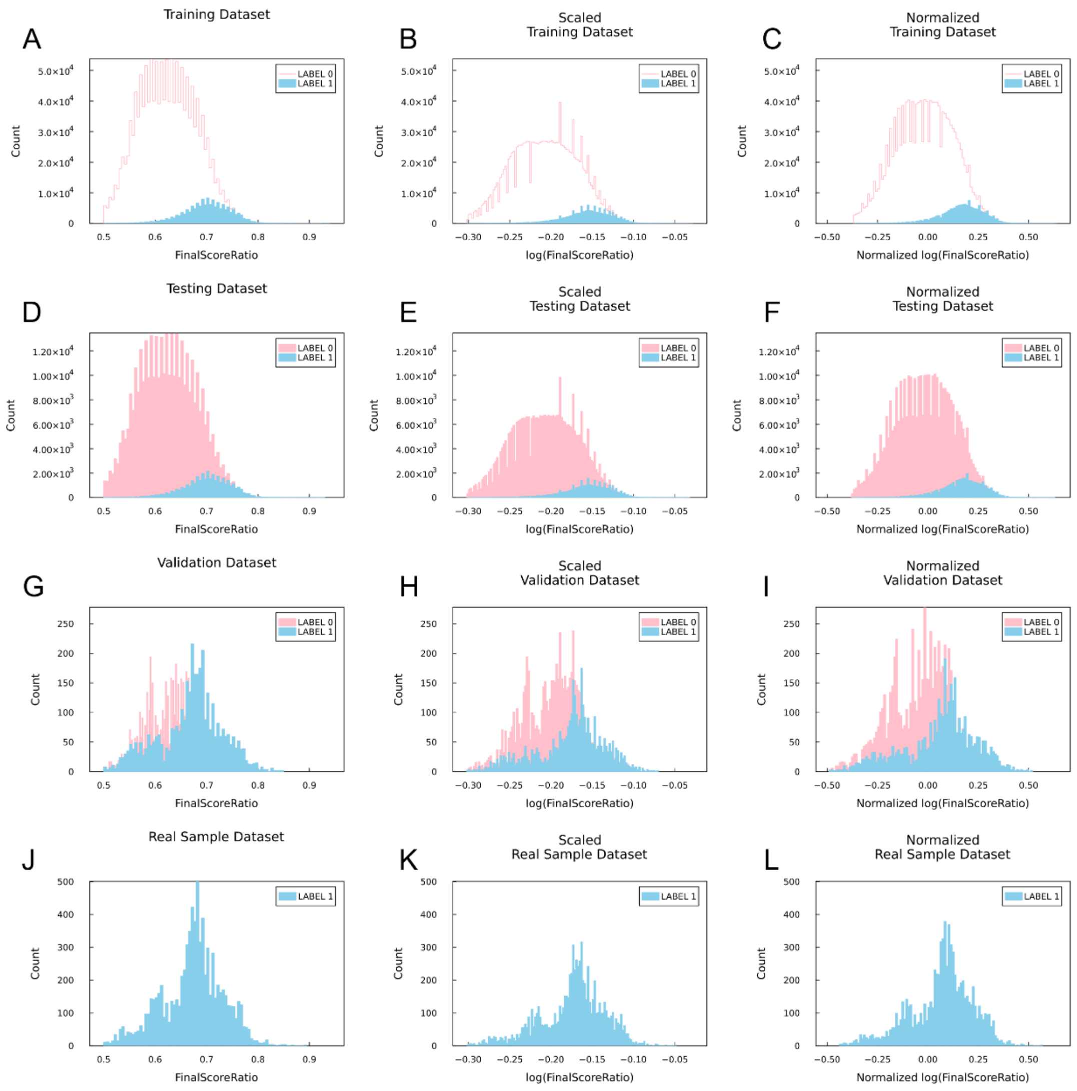

Figure S07: Distribution of the feature "FinalScoreRatio" (S8) in true positives and true negatives data. Histograms illustrating distributions of true positive (LABEL 1) and true negative (LABEL 0) samples for **(A–C)** training, **(D–F)** testing, **(G–I)** validation by pesticides blank spike, and **(J–L)** real sample analyses for the pesticides-spiked black tea sample datasets with the values of S8 were greater than 0.50. Original values (subplots A, D, and G) showed slight separation. Log-transformation (subplots B, E, and H) and normalization by mean (subplots C, F, and I) showed the distributions of data with labels "1" and "0" were slightly split.

Figure S08

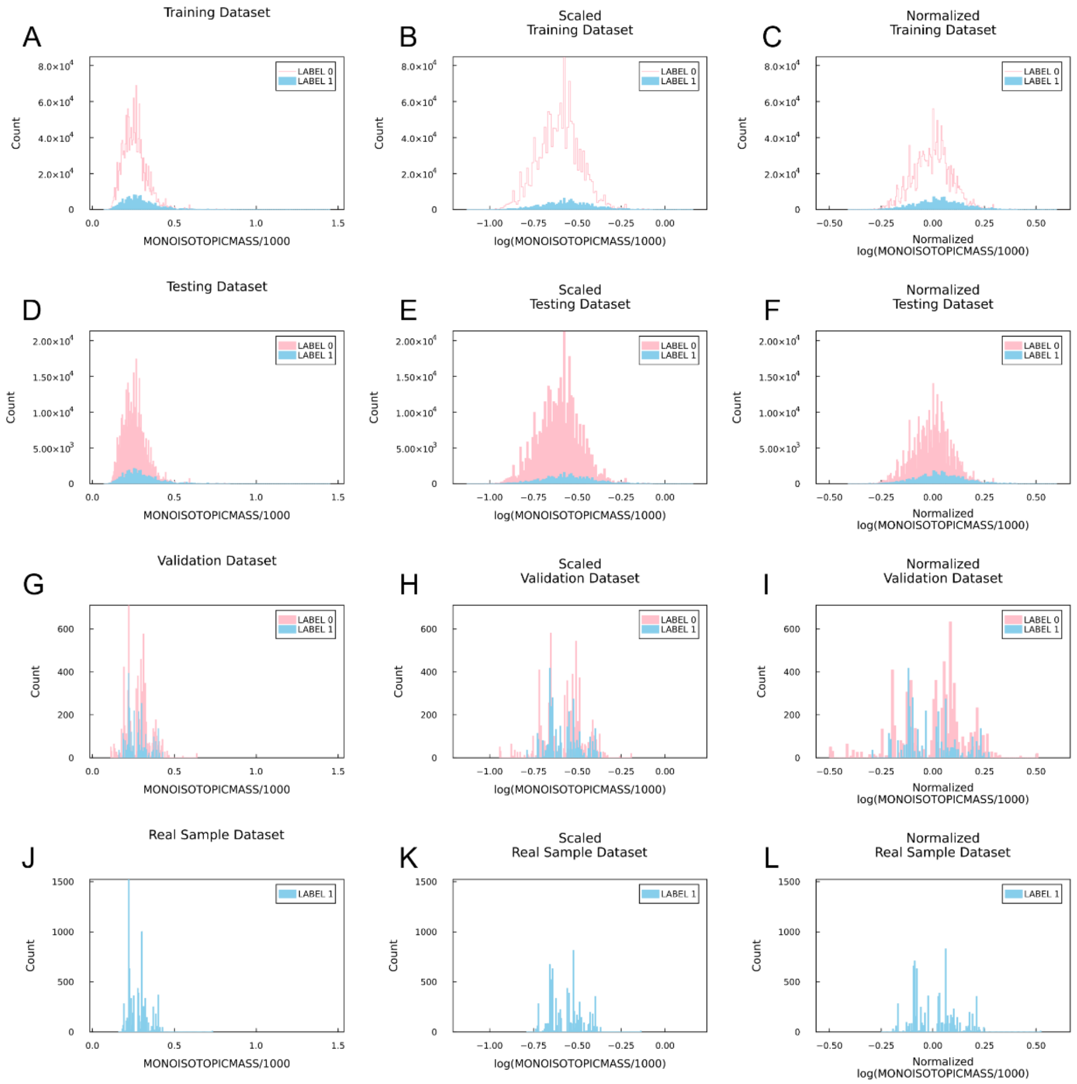

Figure S08: Distribution of the feature "MONOISOTOPICMASS/1000" in true positives and true negatives data. Histograms illustrating distributions of true positive (LABEL 1) and true negative (LABEL 0) samples for **(A–C)** training, **(D–F)** testing, **(G–I)** validation by pesticides blank spike, and **(J–L)** real sample analyses for the pesticides-spiked black tea sample datasets. Original values (subplots A, D, and G) showed right-skewed distributions. Log-transformation (subplots B, E, and H) and normalization by mean (subplots C, F, and I) showed the distributions of data with labels "1" and "0" were slightly split.

Figure S09

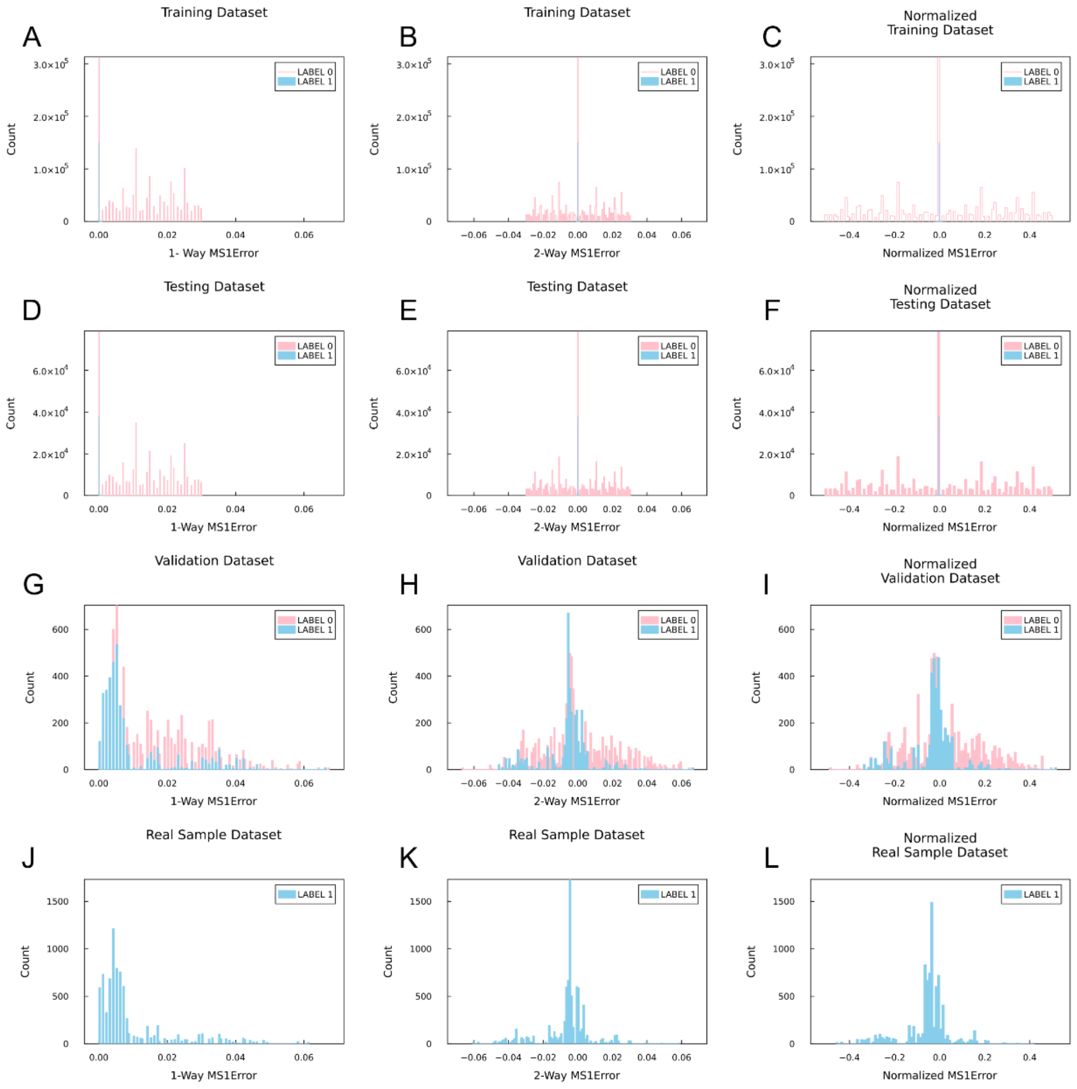

Figure S09: Distribution of the feature "MS1Error" (S3) in true positives and true negatives data. Histograms illustrating distributions of true positive (LABEL 1) and true negative (LABEL 0) samples for **(A–C)** training, **(D–F)** testing, **(G–I)** validation by pesticides blank spike, and **(J–L)** real sample analyses for the pesticides-spiked black tea sample datasets. Absolute values (subplots A, D, G, and J), 2-sided error (subplots B, E, and H) and mean normalized data (subplots C, F, and I) showed the distributions of data with labels "1" and "0" were slightly split.

Figure S10

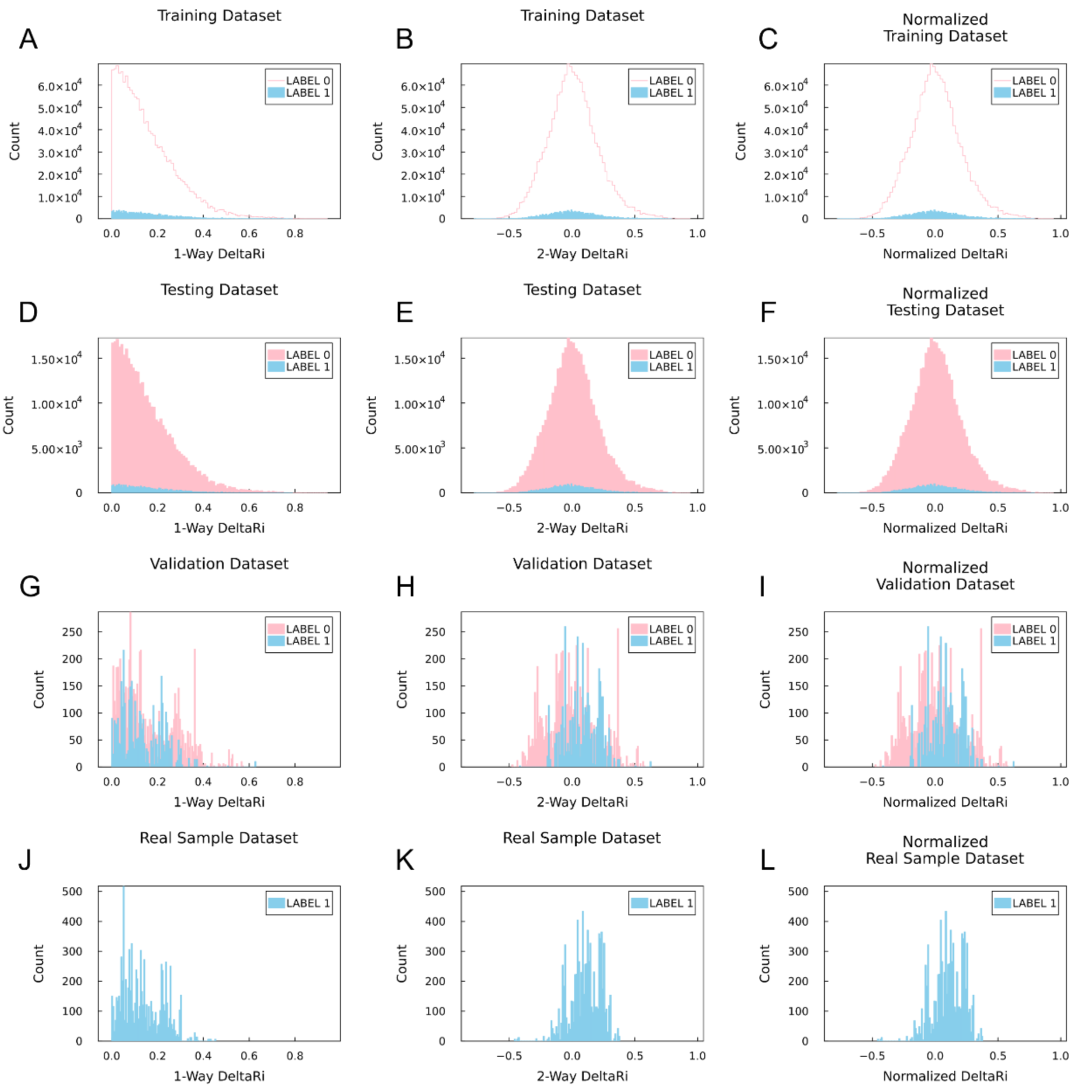

Figure S10: Distribution of the feature "DeltaRi" in true positives and true negatives data. Histograms illustrating distributions of true positive (LABEL 1) and true negative (LABEL 0) samples for **(A–C)** training, **(D–F)** testing, **(G–I)** validation by pesticides blank spike, and **(J–L)** real sample analyses for the pesticides-spiked black tea sample datasets. Absolute values (subplots A, D, and G) showed no separation, while 2-sided retention time index (RTI) error (subplots B, E, and H) and mean normalized data (subplots C, F, and I) showed the distributions of data with labels "1" and "0" were slightly split.

Figure S11

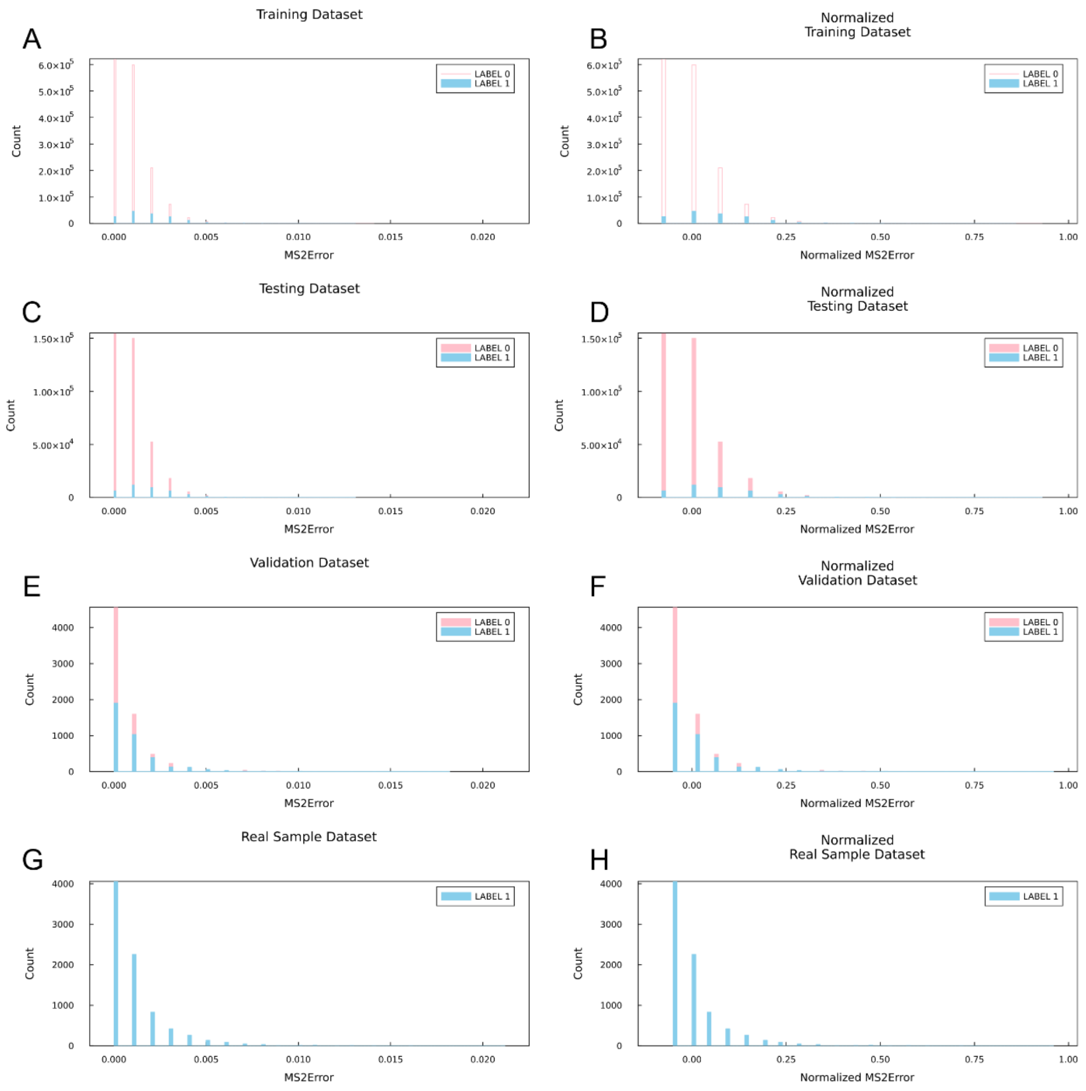

Figure S11: Distribution of the feature "MS2Error" (S4) in true positives and true negatives data. Histograms illustrating distributions of true positive (LABEL 1) and true negative (LABEL 0) samples for **(A–C)** training, **(D–F)** testing, **(G–I)** validation by pesticides blank spike, and **(J–L)** real sample analyses for the pesticides-spiked black tea sample datasets. Absolute values (subplots A, C, and E) and mean normalized data (subplots B, D, and F) showed slight separation in the semi-synthetic data, while no separation of the distributions of data with labels "1" and "0" in the experimental data for validation.

Figure S12

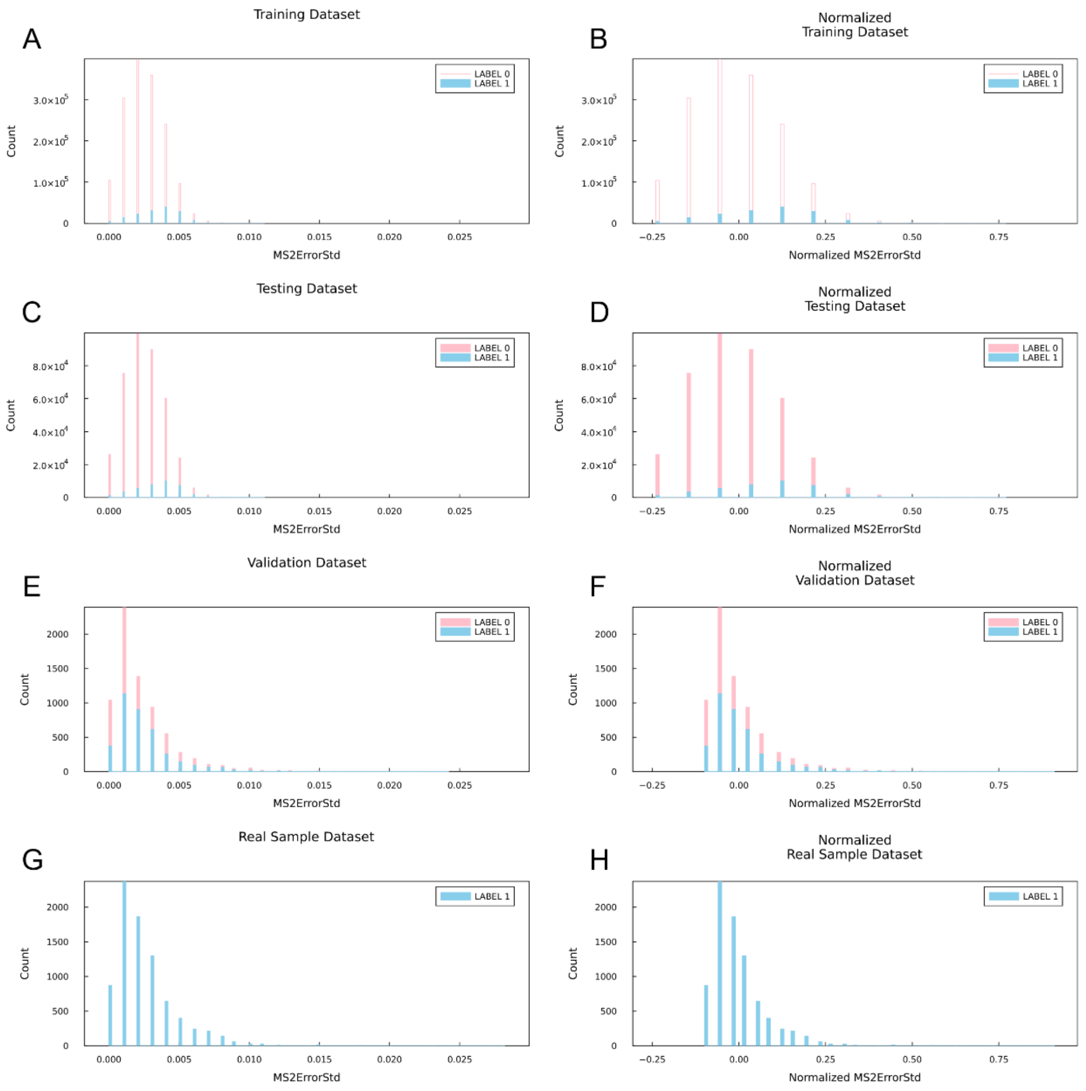

Figure S12: Distribution of the feature "MS2ErrorStd" (S5) in true positives and true negatives data. Histograms illustrating distributions of true positive (LABEL 1) and true negative (LABEL 0) samples for **(A–C)** training, **(D–F)** testing, **(G–I)** validation by pesticides blank spike, and **(J–L)** real sample analyses for the pesticides-spiked black tea sample datasets. Absolute values (subplots A, C, and E) and mean normalized data (subplots B, D, and F) showed slight separation in the semi-synthetic data, while no separation of the distributions of data with labels "1" and "0" in the experimental data for validation.

Figure S13

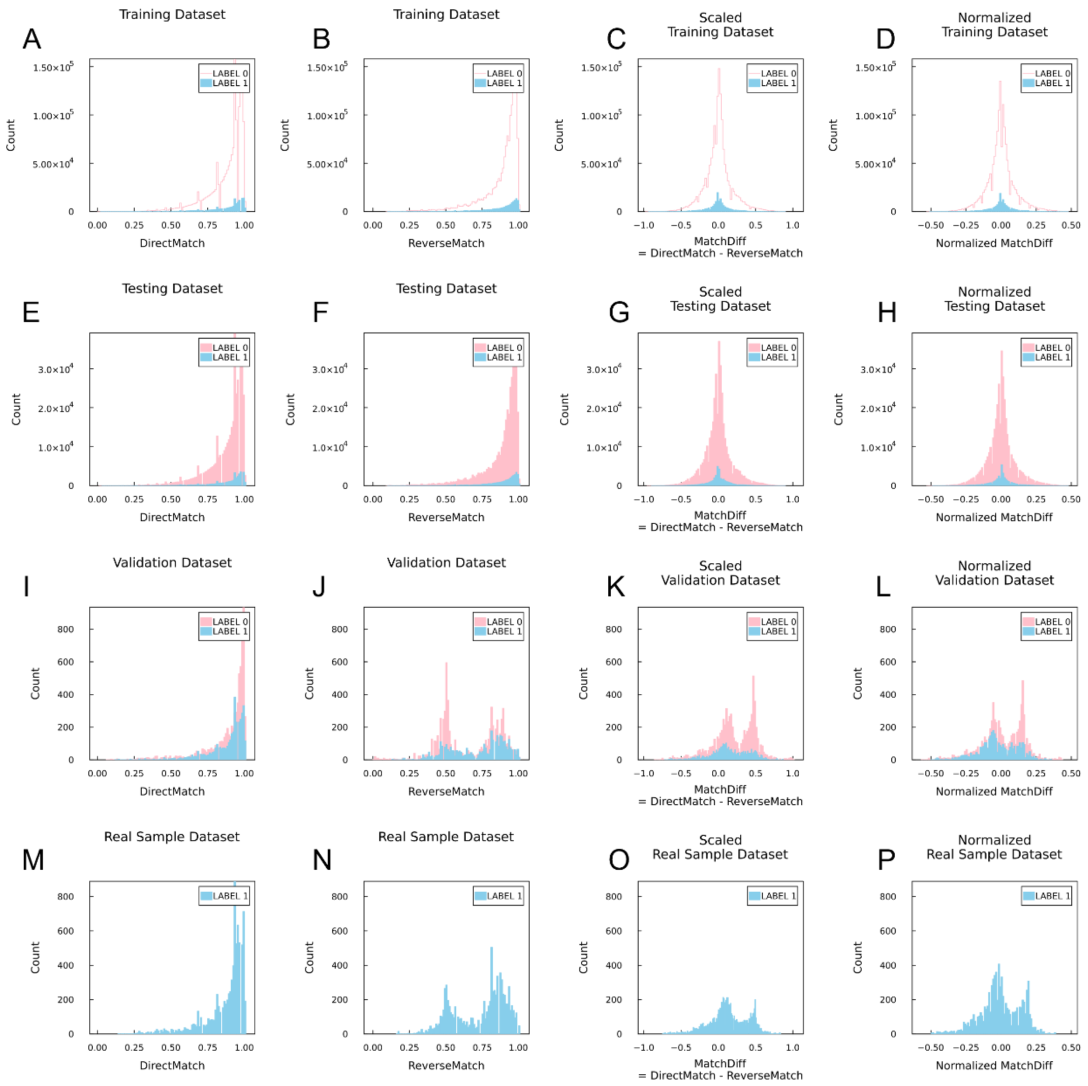

Figure S13: Distribution of the features "DirectMatch" (S6), "ReverseMatch" (S7), and "MatchDiff" in true positives and true negatives data. Histograms illustrating distributions of true positive (LABEL 1) and true negative (LABEL 0) samples for **(A–D)** training, **(E–H)** testing, **(I–L)** validation by pesticides blank spike, and **(M–P)** real sample analyses for the pesticides-spiked black tea sample datasets. Original values (subplots A–B, E–F, I–J, and M–N) showed left-skewed distributions. A new feature "MatchDiff" was created as the difference of the values of S6 and S7. Subtraction of S6 by S7 (subplots C, G, and K) and normalization by mean (subplots D, H, and L) showed the distributions of data with labels "1" and "0" were slightly split.

Figure S14

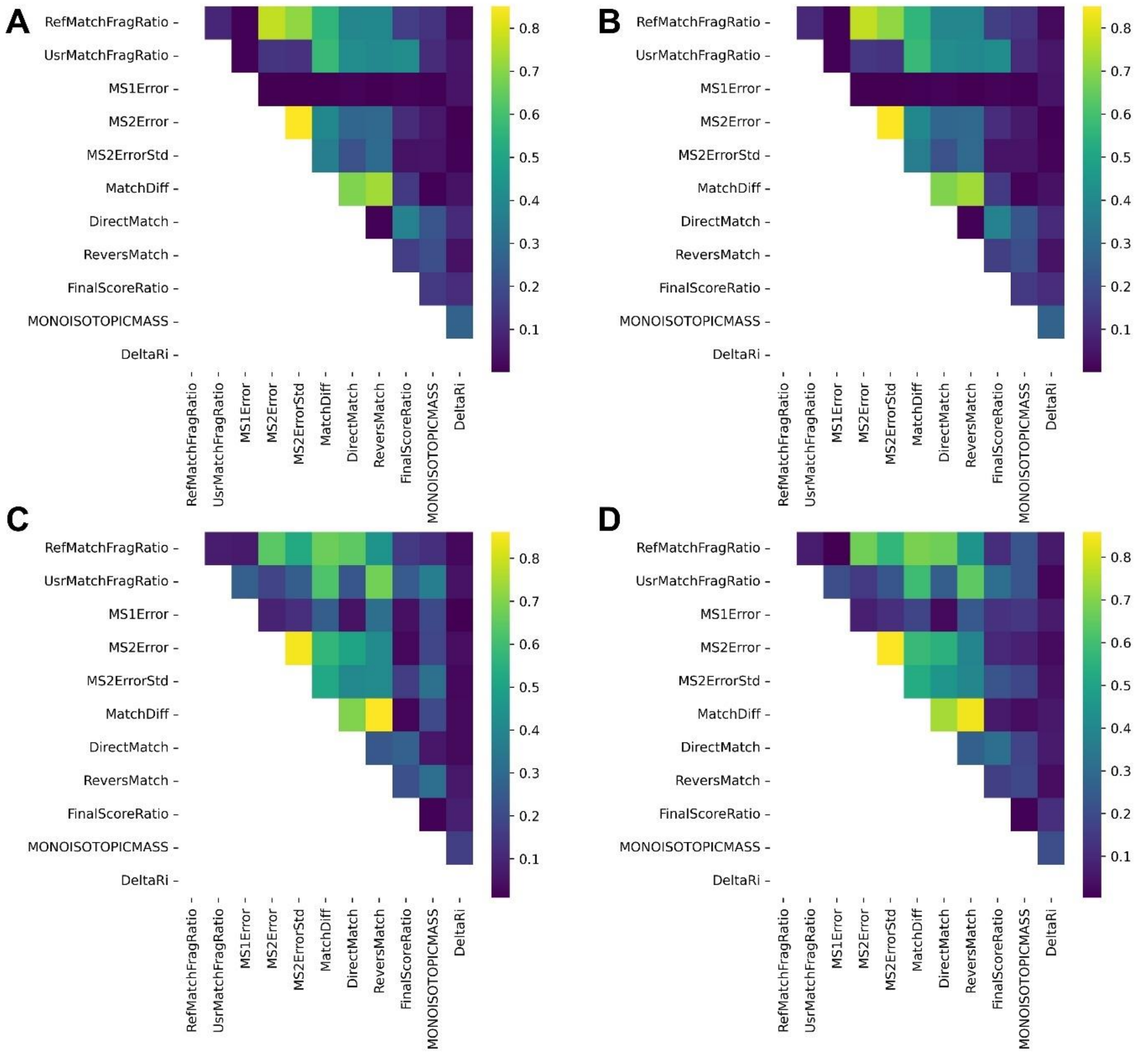

Figure S14: Collinearity assessment of the candidate features in Model 3. Correlation plots for **(A)** training, **(B)** testing, **(C)** pesticides-spiked blank validation, and **(D)** pesticides-spiked real black tea sample datasets.

Figure S15

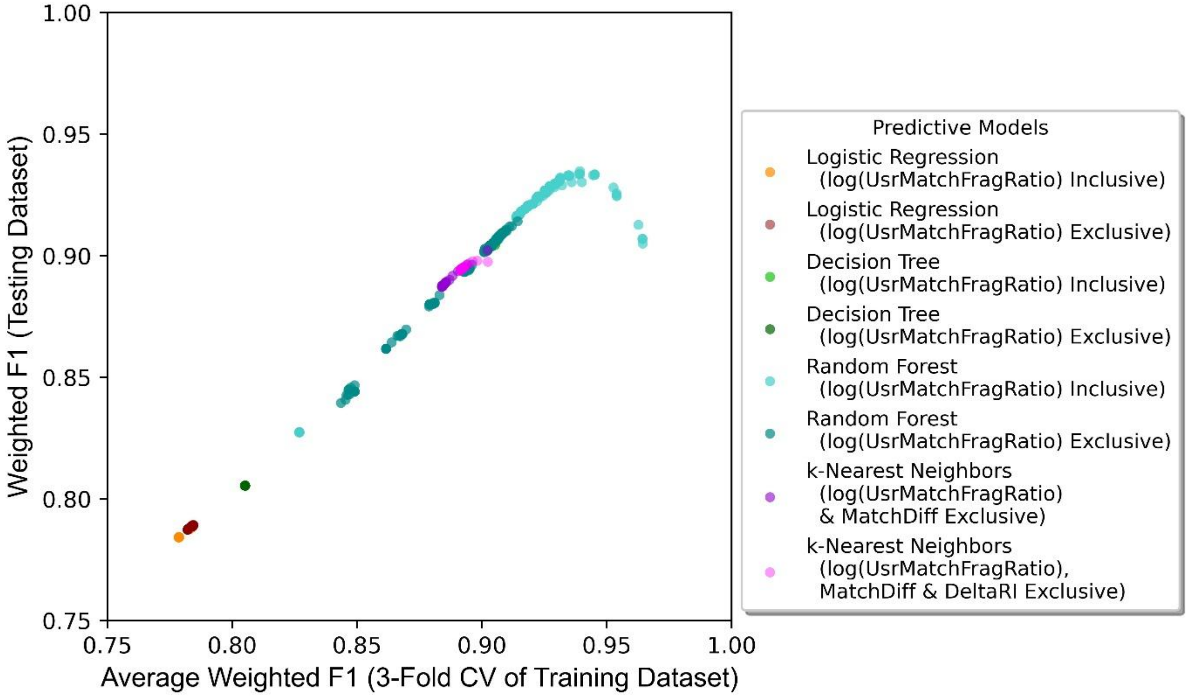

Figure S15: Predictive powers of various machine learning algorithms on semi-synthetic data. Scatter plots illustrate the performance of models attained by different algorithms and hyperparameters. Random forest and k-nearest neighbors models emerged as superior performers compared to decision tree and logistic regression models upon a 3-fold cross-validation (CV).

Figure S16

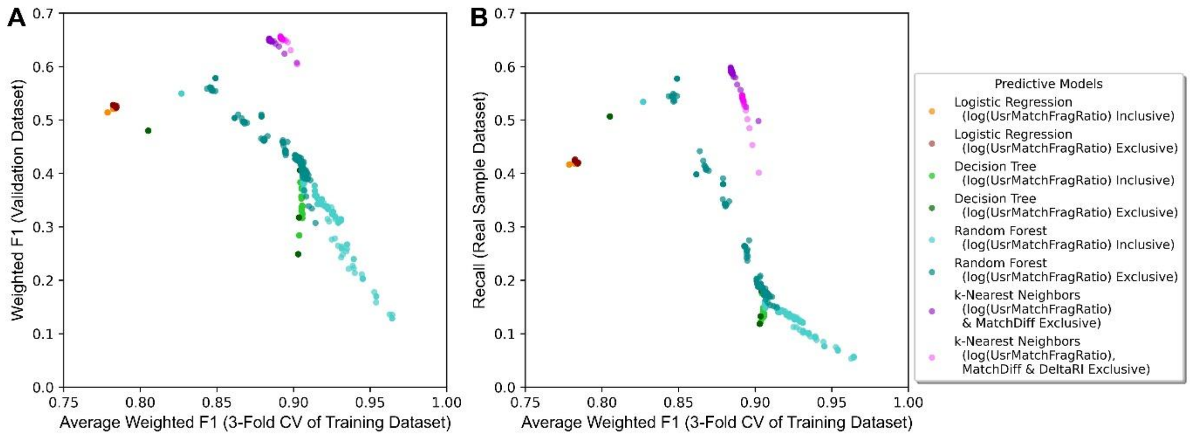

Figure S16: Predictive powers of various machine learning algorithms on experimental data. Scatter plots illustrate the performance of models attained by different algorithms and hyperparameters. **(A)** K-nearest neighbors models showed better performance for the pesticides-spiked blank and **(B)** the tea solution mixture than other models and random forest models. Exclusion of the features "log(UsrMatchFragRatio)" and "MatchDiff" in the KNN models exhibited better recalls than the models without the feature "DeltaRi".

ADDITIONAL TABLES

Table S1: Computational Power and Packages Used for Modeling.

Workstation	
Edition	Windows 11 Pro
Version	23H2
Installed on	11/10/2023
OS build	22631.4169
Experience	Windows Feature Experience Pack 1000.22700.1034.0
Processor	12th Gen Intel(R) Core(TM) i7-12700K 3.60 GHz
Installed RAM	192 GB (192 GB usable)
System type	64-bit operating system, x64-based processor

Programming languages	Packages
Julia	Pkg, BSON, CSV, DataFrames, PyCall, LinearAlgebra, Statistics, ScikitLearn, Random, ProgressBars, StatsPlots, Plots, Conda (pubchempy, padelpy, joblib)
Python	xlwings , pandas, matplotlib, numpy, statistics, numpy, seaborn, sklearn

Table S2: NTA Data Pre-Processing by jHRMS ToolBox.

	Module	Function / Definition	Default	Data Type
	**MS_Import.jl**	**LC/HRMS file format conversion**		
Input Para-meters	pathin	the path to the folder with the files to be imported		**String**
	filenames	the file name including the extension .mzXML		**String**
	mz_thresh	the mass window to be imported	[0,0]	**1x2 Array{Int64,2}**
	int_thersh	the minimum absolute intensity for the datapoint to be imported	0	**Int64**
	**SAFD.jl**	**LC-MS/MS features extraction**		
Input Para-meters	mz_vals	an m x n matrix of the measured m/z values with m being the scan numbers and n being the mass channels		
	mz_int	an m x n matrix of intensities measured for each m/z values with m being the scan numbers and n being the mass channels		
	Rt	a vector of RT		**1xn Array{Float64,n}**
	FileName	the name of the file		**String**
	path	the path to the folder where the final report (a CSV file) will be saved		**String**
	max_numb_iter	the maximum number of iterations	20000	**Int64**
	max_t_peak_w	the maximum number of scans within a chromatographic feature	30	**Int64**
	res	the expected mass resolution	20000	**Int64**
	min_ms_w	the minimum peak width in the mass domain	0.01	**Float64**
	r_thresh	the R2 threshold for the Gaussian fit	0.75	**Float64**
	min_int	the minimum intensity for the features to be detected	1000	**Int64**
	sig_inc_thresh	the detection of the overlapping features in the time domain	5	**Int64**
	S2N	the S/N for feature filtering	2	**Int64**
	min_peak_w_s	the second threshold for feature filtering	3	**Int64**
	**CompCreate.jl**	**Componentization**		
Input Para-meters	chrom	all the relevant information related to LC-MS run extracted from the mzXML file		**Dict{Any}**
	path2features	the path to a CSV file that contains the feature list from SAFD.jl module		**String**
	mass_win_per	the percentage of the peak within the mass domain used for the calculation of mass tolerance	0.75	**Float64**
	ret_win_per	the percentage of the peak within the time domain used for the calculation of RT tolerance	0.5	**Float64**
	r_thresh	the correlation coefficient threshold used during the comparison of the peak shape of the parent ion and the fragments	0.85	**Float64**

	delta_mass	the KMD deviation used for the detection of isotopes	0.004	**Float64**
	**ulsa.jl**	**Compound annotation**		
Input Para-meters	mode	the ionization mode selected for MS data acquistion	"POSITIVE"	**String**
	source	the ion source	"ESI"	**String**
	path2comp	the path to a CSV file that contains the components from CompCreate.jl module		**String**
	weight_f	an array of 7 weighting factors, values ranging between 0 and 1	[1,1,1,1,1,1,1]	**Array{Any}**

Table S3: Hyper-parameters Optimization History for Model 2.

min_samples _leaf	n_ estimators	R^2_ Training Set	R^2_ 90% Split Validation Set	Avg. R^2_ n-Fold CV	R^2_ 10 % Split Testing Set	state	n-Fold CV
4	300	0.8491	0.8825	0.6295	0.7812	42	3
4	250	0.8485	0.8817	0.6294	0.7807	42	3
6	250	0.8043	0.8308	0.6230	0.7508	42	3
6	200	0.8040	0.8309	0.6230	0.7493	42	3
6	200	0.8043	0.8320	0.6230	0.7479	42	3
6	150	0.8034	0.8307	0.6226	0.7479	42	3
6	150	0.8038	0.8317	0.6226	0.7468	42	3
8	100	0.7703	0.7940	0.6163	0.7219	42	3
8	100	0.7702	0.7945	0.6163	0.7206	42	3
2	1000	0.9045	0.9465	0.6350	0.8193	42	3
2	600	0.9040	0.9460	0.6348	0.8193	42	3
2	500	0.9043	0.9471	0.6347	0.8172	42	3
2	400	0.9048	0.9464	0.6346	0.8199	42	3
2	400	0.9044	0.9464	0.6346	0.8191	42	3
2	400	0.9041	0.9464	0.6346	0.8184	42	3
2	400	0.9038	0.9466	0.6346	0.8168	42	3
2	350	0.9043	0.9469	0.6346	0.8178	42	3
2	400	0.9039	0.9464	0.6345	0.8177	42	3
2	350	0.9041	0.9461	0.6345	0.8193	42	3
2	200	0.9043	0.9466	0.6341	0.8181	42	3
2	150	0.9046	0.9457	0.6337	0.8213	42	3
2	150	0.9040	0.9456	0.6337	0.8197	42	3
2	100	0.9028	0.9453	0.6325	0.8167	42	3
1	1000	0.9290	0.9761	0.6321	0.8326	42	3
1	350	0.9286	0.9758	0.6317	0.8322	42	3
1	350	0.9279	0.9757	0.6317	0.8309	42	3
1	400	0.9285	0.9759	0.6316	0.8321	42	3
1	200	0.9277	0.9755	0.6311	0.8307	42	3
1	150	0.9268	0.9751	0.6301	0.8295	42	3
4	400	0.8493	0.8819	0.6296	0.7835	42	3
4	350	0.8486	0.8830	0.6296	0.7790	42	3
4	350	0.8481	0.8826	0.6296	0.7781	42	3
2	50	0.9013	0.9441	0.6294	0.8139	42	3
4	250	0.8481	0.8824	0.6294	0.7786	42	3
1	100	0.9270	0.9749	0.6294	0.8291	42	3
4	100	0.8473	0.8814	0.6279	0.7778	42	3
4	50	0.8466	0.8803	0.6257	0.7779	42	3
6	350	0.8042	0.8321	0.6234	0.7476	42	3
6	400	0.8045	0.8322	0.6234	0.7484	42	3
6	300	0.8038	0.8321	0.6232	0.7462	42	3
6	250	0.8043	0.8323	0.6230	0.7472	42	3
6	250	0.8040	0.8323	0.6230	0.7469	42	3

6	150	0.8032	0.8303	0.6226	0.7479	42	3
8	300	0.7717	0.7943	0.6172	0.7263	42	3
8	200	0.7713	0.7947	0.6170	0.7236	42	3
8	200	0.7713	0.7947	0.6170	0.7233	42	3
8	150	0.7715	0.7937	0.6168	0.7266	42	3
8	150	0.7711	0.7938	0.6168	0.7250	42	3
8	100	0.7704	0.7936	0.6163	0.7231	42	3
1	350	0.9669	0.9791	0.6464	0.8585	42	10
1	350	0.9668	0.9793	0.6462	0.8553	1	10
2	250	0.9430	0.9534	0.6492	0.8483	42	10
2	250	0.9428	0.9535	0.6489	0.8455	1	10
4	200	0.8891	0.8976	0.6437	0.8113	1	10
4	200	0.8887	0.8977	0.6438	0.8077	42	10
4	50	0.8868	0.8959	0.6403	0.8051	1	10
4	50	0.8864	0.8959	0.6403	0.8012	42	10

Table S4: Hyper-parameters Optimization History for Model 3.

n_ neighbors	leaf_ size	weights	metric	p	wF1_ train	wMCC_ train	wF1_ test	wMCC_ test	Avg. wF1_ 3-Fold CV	wF1_ spike Blank	wMCC_ spike Blank	recall_ real
10	10	distance	minkowski	2	1.00	1.00	0.92	0.83	0.93	0.50	0.17	0.45
10	10	distance	euclidean	1	1.00	1.00	0.92	0.83	0.93	0.50	0.17	0.45
10	10	distance	euclidean	2	1.00	1.00	0.92	0.83	0.93	0.50	0.17	0.45
10	1	distance	minkowski	2	1.00	1.00	0.92	0.83	0.93	0.50	0.17	0.45
10	1	distance	euclidean	1	1.00	1.00	0.92	0.83	0.93	0.50	0.17	0.45
10	1	distance	euclidean	2	1.00	1.00	0.92	0.83	0.93	0.50	0.17	0.45
10	5	distance	minkowski	2	1.00	1.00	0.92	0.83	0.93	0.50	0.17	0.45
10	5	distance	euclidean	1	1.00	1.00	0.92	0.83	0.93	0.50	0.17	0.45
10	5	distance	euclidean	2	1.00	1.00	0.92	0.83	0.93	0.50	0.17	0.45
10	2	distance	minkowski	2	1.00	1.00	0.92	0.83	0.93	0.50	0.17	0.45
10	2	distance	euclidean	1	1.00	1.00	0.92	0.83	0.93	0.50	0.17	0.45
10	2	distance	euclidean	2	1.00	1.00	0.92	0.83	0.93	0.50	0.17	0.45
10	10	uniform	minkowski	2	0.96	0.92	0.91	0.82	0.93	0.50	0.17	0.45
10	10	uniform	euclidean	1	0.96	0.92	0.91	0.82	0.93	0.50	0.17	0.45
10	10	uniform	euclidean	2	0.96	0.92	0.91	0.82	0.93	0.50	0.17	0.45
10	1	uniform	minkowski	2	0.96	0.92	0.91	0.82	0.93	0.50	0.17	0.45
10	1	uniform	euclidean	1	0.96	0.92	0.91	0.82	0.93	0.50	0.17	0.45
10	1	uniform	euclidean	2	0.96	0.92	0.91	0.82	0.93	0.50	0.17	0.45
10	5	uniform	minkowski	2	0.96	0.92	0.91	0.82	0.93	0.50	0.17	0.45
10	5	uniform	euclidean	1	0.96	0.92	0.91	0.82	0.93	0.50	0.17	0.45
10	5	uniform	euclidean	2	0.96	0.92	0.91	0.82	0.93	0.50	0.17	0.45
10	2	uniform	minkowski	2	0.96	0.92	0.91	0.82	0.93	0.50	0.17	0.45
10	2	uniform	euclidean	1	0.96	0.92	0.91	0.82	0.93	0.50	0.17	0.45
10	2	uniform	euclidean	2	0.96	0.92	0.91	0.82	0.93	0.50	0.17	0.45
10	1	uniform	minkowski	1	0.96	0.93	0.92	0.85	0.93	0.51	0.17	0.45
10	1	uniform	manhattan	1	0.96	0.93	0.92	0.85	0.93	0.51	0.17	0.45
10	1	uniform	manhattan	2	0.96	0.93	0.92	0.85	0.93	0.51	0.17	0.45
10	2	uniform	minkowski	1	0.96	0.93	0.92	0.85	0.93	0.51	0.17	0.45
10	2	uniform	manhattan	1	0.96	0.93	0.92	0.85	0.93	0.51	0.17	0.45
10	2	uniform	manhattan	2	0.96	0.93	0.92	0.85	0.93	0.51	0.17	0.45
10	5	uniform	minkowski	1	0.96	0.93	0.92	0.85	0.93	0.51	0.17	0.45
10	5	uniform	manhattan	1	0.96	0.93	0.92	0.85	0.93	0.51	0.17	0.45
10	5	uniform	manhattan	2	0.96	0.93	0.92	0.85	0.93	0.51	0.17	0.45
10	10	uniform	minkowski	1	0.96	0.93	0.92	0.85	0.93	0.51	0.17	0.45
10	10	uniform	manhattan	1	0.96	0.93	0.92	0.85	0.93	0.51	0.17	0.45
10	10	uniform	manhattan	2	0.96	0.93	0.92	0.85	0.93	0.51	0.17	0.45
10	10	distance	minkowski	1	1.00	1.00	0.93	0.85	0.93	0.51	0.17	0.45
10	10	distance	manhattan	1	1.00	1.00	0.93	0.85	0.93	0.51	0.17	0.45
10	10	distance	manhattan	2	1.00	1.00	0.93	0.85	0.93	0.51	0.17	0.45
10	1	distance	minkowski	1	1.00	1.00	0.93	0.85	0.93	0.51	0.17	0.45
10	1	distance	manhattan	1	1.00	1.00	0.93	0.85	0.93	0.51	0.17	0.45
10	1	distance	manhattan	2	1.00	1.00	0.93	0.85	0.93	0.51	0.17	0.45

10	2	distance	minkowski	1	1.00	1.00	0.93	0.85	0.93	0.51	0.17	0.45
10	2	distance	manhattan	1	1.00	1.00	0.93	0.85	0.93	0.51	0.17	0.45
10	2	distance	manhattan	2	1.00	1.00	0.93	0.85	0.93	0.51	0.17	0.45
10	5	distance	minkowski	1	1.00	1.00	0.93	0.85	0.93	0.51	0.17	0.45
10	5	distance	manhattan	1	1.00	1.00	0.93	0.85	0.93	0.51	0.17	0.45
10	5	distance	manhattan	2	1.00	1.00	0.93	0.85	0.93	0.51	0.17	0.45
5	10	uniform	minkowski	1	0.98	0.96	0.92	0.84	0.94	0.43	0.13	0.37
5	10	uniform	manhattan	1	0.98	0.96	0.92	0.84	0.94	0.43	0.13	0.37
5	10	uniform	manhattan	2	0.98	0.96	0.92	0.84	0.94	0.43	0.13	0.37
5	1	uniform	minkowski	1	0.98	0.96	0.92	0.84	0.94	0.43	0.13	0.37
5	1	uniform	manhattan	1	0.98	0.96	0.92	0.84	0.94	0.43	0.13	0.37
5	1	uniform	manhattan	2	0.98	0.96	0.92	0.84	0.94	0.43	0.13	0.37
5	2	uniform	minkowski	1	0.98	0.96	0.92	0.84	0.94	0.43	0.13	0.37
5	2	uniform	manhattan	1	0.98	0.96	0.92	0.84	0.94	0.43	0.13	0.37
5	2	uniform	manhattan	2	0.98	0.96	0.92	0.84	0.94	0.43	0.13	0.37
5	5	uniform	minkowski	1	0.98	0.96	0.92	0.84	0.94	0.43	0.13	0.37
5	5	uniform	manhattan	1	0.98	0.96	0.92	0.84	0.94	0.43	0.13	0.37
5	5	uniform	manhattan	2	0.98	0.96	0.92	0.84	0.94	0.43	0.13	0.37
5	10	distance	minkowski	1	1.00	1.00	0.92	0.84	0.94	0.43	0.13	0.37
5	10	distance	manhattan	1	1.00	1.00	0.92	0.84	0.94	0.43	0.13	0.37
5	10	distance	manhattan	2	1.00	1.00	0.92	0.84	0.94	0.43	0.13	0.37
5	1	distance	minkowski	1	1.00	1.00	0.92	0.84	0.94	0.43	0.13	0.37
5	1	distance	manhattan	1	1.00	1.00	0.92	0.84	0.94	0.43	0.13	0.37
5	1	distance	manhattan	2	1.00	1.00	0.92	0.84	0.94	0.43	0.13	0.37
5	2	distance	minkowski	1	1.00	1.00	0.92	0.84	0.94	0.43	0.13	0.37
5	2	distance	manhattan	1	1.00	1.00	0.92	0.84	0.94	0.43	0.13	0.37
5	2	distance	manhattan	2	1.00	1.00	0.92	0.84	0.94	0.43	0.13	0.37
5	5	distance	minkowski	1	1.00	1.00	0.92	0.84	0.94	0.43	0.13	0.37
5	5	distance	manhattan	1	1.00	1.00	0.92	0.84	0.94	0.43	0.13	0.37
5	5	distance	manhattan	2	1.00	1.00	0.92	0.84	0.94	0.43	0.13	0.37
5	1	uniform	minkowski	2	0.98	0.95	0.90	0.81	0.94	0.44	0.14	0.37
5	1	uniform	euclidean	1	0.98	0.95	0.90	0.81	0.94	0.44	0.14	0.37
5	1	uniform	euclidean	2	0.98	0.95	0.90	0.81	0.94	0.44	0.14	0.37
5	2	uniform	minkowski	2	0.98	0.95	0.90	0.81	0.94	0.44	0.14	0.37
5	2	uniform	euclidean	1	0.98	0.95	0.90	0.81	0.94	0.44	0.14	0.37
5	2	uniform	euclidean	2	0.98	0.95	0.90	0.81	0.94	0.44	0.14	0.37
5	5	uniform	minkowski	2	0.98	0.95	0.90	0.81	0.94	0.44	0.14	0.37
5	5	uniform	euclidean	1	0.98	0.95	0.90	0.81	0.94	0.44	0.14	0.37
5	5	uniform	euclidean	2	0.98	0.95	0.90	0.81	0.94	0.44	0.14	0.37
5	10	uniform	minkowski	2	0.98	0.95	0.90	0.81	0.94	0.44	0.14	0.37
5	10	uniform	euclidean	1	0.98	0.95	0.90	0.81	0.94	0.44	0.14	0.37
5	10	uniform	euclidean	2	0.98	0.95	0.90	0.81	0.94	0.44	0.14	0.37
5	1	distance	minkowski	2	1.00	1.00	0.90	0.81	0.94	0.44	0.14	0.37
5	1	distance	euclidean	1	1.00	1.00	0.90	0.81	0.94	0.44	0.14	0.37
5	1	distance	euclidean	2	1.00	1.00	0.90	0.81	0.94	0.44	0.14	0.37
5	2	distance	minkowski	2	1.00	1.00	0.90	0.81	0.94	0.44	0.14	0.37

5	2	distance	euclidean	1	1.00	1.00	0.90	0.81	0.94	0.44	0.14	0.37
5	2	distance	euclidean	2	1.00	1.00	0.90	0.81	0.94	0.44	0.14	0.37
5	5	distance	minkowski	2	1.00	1.00	0.90	0.81	0.94	0.44	0.14	0.37
5	5	distance	euclidean	1	1.00	1.00	0.90	0.81	0.94	0.44	0.14	0.37
5	5	distance	euclidean	2	1.00	1.00	0.90	0.81	0.94	0.44	0.14	0.37
5	10	distance	minkowski	2	1.00	1.00	0.90	0.81	0.94	0.44	0.14	0.37
5	10	distance	euclidean	1	1.00	1.00	0.90	0.81	0.94	0.44	0.14	0.37
5	10	distance	euclidean	2	1.00	1.00	0.90	0.81	0.94	0.44	0.14	0.37
2	1	uniform	minkowski	2	1.00	1.00	0.84	0.74	0.97	0.30	0.09	0.22
2	1	uniform	euclidean	1	1.00	1.00	0.84	0.74	0.97	0.30	0.09	0.22
2	1	uniform	euclidean	2	1.00	1.00	0.84	0.74	0.97	0.30	0.09	0.22
2	1	distance	minkowski	2	1.00	1.00	0.84	0.74	0.97	0.30	0.09	0.22
2	1	distance	euclidean	1	1.00	1.00	0.84	0.74	0.97	0.30	0.09	0.22
2	1	distance	euclidean	2	1.00	1.00	0.84	0.74	0.97	0.30	0.09	0.22
2	2	uniform	minkowski	2	1.00	1.00	0.84	0.74	0.97	0.30	0.09	0.22
2	2	uniform	euclidean	1	1.00	1.00	0.84	0.74	0.97	0.30	0.09	0.22
2	2	uniform	euclidean	2	1.00	1.00	0.84	0.74	0.97	0.30	0.09	0.22
2	2	distance	minkowski	2	1.00	1.00	0.84	0.74	0.97	0.30	0.09	0.22
2	2	distance	euclidean	1	1.00	1.00	0.84	0.74	0.97	0.30	0.09	0.22
2	2	distance	euclidean	2	1.00	1.00	0.84	0.74	0.97	0.30	0.09	0.22
2	5	uniform	minkowski	2	1.00	1.00	0.84	0.74	0.97	0.30	0.09	0.22
2	5	uniform	euclidean	1	1.00	1.00	0.84	0.74	0.97	0.30	0.09	0.22
2	5	uniform	euclidean	2	1.00	1.00	0.84	0.74	0.97	0.30	0.09	0.22
2	5	distance	minkowski	2	1.00	1.00	0.84	0.74	0.97	0.30	0.09	0.22
2	5	distance	euclidean	1	1.00	1.00	0.84	0.74	0.97	0.30	0.09	0.22
2	5	distance	euclidean	2	1.00	1.00	0.84	0.74	0.97	0.30	0.09	0.22
2	10	uniform	minkowski	2	1.00	1.00	0.84	0.74	0.97	0.30	0.09	0.22
2	10	uniform	euclidean	1	1.00	1.00	0.84	0.74	0.97	0.30	0.09	0.22
2	10	uniform	euclidean	2	1.00	1.00	0.84	0.74	0.97	0.30	0.09	0.22
2	10	distance	minkowski	2	1.00	1.00	0.84	0.74	0.97	0.30	0.09	0.22
2	10	distance	euclidean	1	1.00	1.00	0.84	0.74	0.97	0.30	0.09	0.22
2	10	distance	euclidean	2	1.00	1.00	0.84	0.74	0.97	0.30	0.09	0.22
1	2	uniform	minkowski	2	1.00	1.00	0.84	0.74	0.97	0.30	0.09	0.22
1	2	uniform	euclidean	1	1.00	1.00	0.84	0.74	0.97	0.30	0.09	0.22
1	2	uniform	euclidean	2	1.00	1.00	0.84	0.74	0.97	0.30	0.09	0.22
1	2	distance	minkowski	2	1.00	1.00	0.84	0.74	0.97	0.30	0.09	0.22
1	2	distance	euclidean	1	1.00	1.00	0.84	0.74	0.97	0.30	0.09	0.22
1	2	distance	euclidean	2	1.00	1.00	0.84	0.74	0.97	0.30	0.09	0.22
1	5	uniform	minkowski	2	1.00	1.00	0.84	0.74	0.97	0.30	0.09	0.22
1	5	uniform	euclidean	1	1.00	1.00	0.84	0.74	0.97	0.30	0.09	0.22
1	5	uniform	euclidean	2	1.00	1.00	0.84	0.74	0.97	0.30	0.09	0.22
1	5	distance	minkowski	2	1.00	1.00	0.84	0.74	0.97	0.30	0.09	0.22
1	5	distance	euclidean	1	1.00	1.00	0.84	0.74	0.97	0.30	0.09	0.22
1	5	distance	euclidean	2	1.00	1.00	0.84	0.74	0.97	0.30	0.09	0.22
1	1	uniform	minkowski	2	1.00	1.00	0.84	0.74	0.97	0.30	0.09	0.22
1	1	uniform	euclidean	1	1.00	1.00	0.84	0.74	0.97	0.30	0.09	0.22

1	1	uniform	euclidean	2	1.00	1.00	0.84	0.74	0.97	0.30	0.09	0.22
1	1	distance	minkowski	2	1.00	1.00	0.84	0.74	0.97	0.30	0.09	0.22
1	1	distance	euclidean	1	1.00	1.00	0.84	0.74	0.97	0.30	0.09	0.22
1	1	distance	euclidean	2	1.00	1.00	0.84	0.74	0.97	0.30	0.09	0.22
1	10	uniform	minkowski	2	1.00	1.00	0.84	0.74	0.97	0.30	0.09	0.22
1	10	uniform	euclidean	1	1.00	1.00	0.84	0.74	0.97	0.30	0.09	0.22
1	10	uniform	euclidean	2	1.00	1.00	0.84	0.74	0.97	0.30	0.09	0.22
1	10	distance	minkowski	2	1.00	1.00	0.84	0.74	0.97	0.30	0.09	0.22
1	10	distance	euclidean	1	1.00	1.00	0.84	0.74	0.97	0.30	0.09	0.22
1	10	distance	euclidean	2	1.00	1.00	0.84	0.74	0.97	0.30	0.09	0.22
2	1	uniform	minkowski	1	1.00	1.00	0.86	0.77	0.97	0.30	0.10	0.22
2	1	uniform	manhattan	1	1.00	1.00	0.86	0.77	0.97	0.30	0.10	0.22
2	1	uniform	manhattan	2	1.00	1.00	0.86	0.77	0.97	0.30	0.10	0.22
2	1	distance	minkowski	1	1.00	1.00	0.86	0.77	0.97	0.30	0.10	0.22
2	1	distance	manhattan	1	1.00	1.00	0.86	0.77	0.97	0.30	0.10	0.22
2	1	distance	manhattan	2	1.00	1.00	0.86	0.77	0.97	0.30	0.10	0.22
2	2	uniform	minkowski	1	1.00	1.00	0.86	0.77	0.97	0.30	0.10	0.22
2	2	uniform	manhattan	1	1.00	1.00	0.86	0.77	0.97	0.30	0.10	0.22
2	2	uniform	manhattan	2	1.00	1.00	0.86	0.77	0.97	0.30	0.10	0.22
2	2	distance	minkowski	1	1.00	1.00	0.86	0.77	0.97	0.30	0.10	0.22
2	2	distance	manhattan	1	1.00	1.00	0.86	0.77	0.97	0.30	0.10	0.22
2	2	distance	manhattan	2	1.00	1.00	0.86	0.77	0.97	0.30	0.10	0.22
2	5	uniform	minkowski	1	1.00	1.00	0.86	0.77	0.97	0.30	0.10	0.22
2	5	uniform	manhattan	1	1.00	1.00	0.86	0.77	0.97	0.30	0.10	0.22
2	5	uniform	manhattan	2	1.00	1.00	0.86	0.77	0.97	0.30	0.10	0.22
2	5	distance	minkowski	1	1.00	1.00	0.86	0.77	0.97	0.30	0.10	0.22
2	5	distance	manhattan	1	1.00	1.00	0.86	0.77	0.97	0.30	0.10	0.22
2	5	distance	manhattan	2	1.00	1.00	0.86	0.77	0.97	0.30	0.10	0.22
2	10	uniform	minkowski	1	1.00	1.00	0.86	0.77	0.97	0.30	0.10	0.22
2	10	uniform	manhattan	1	1.00	1.00	0.86	0.77	0.97	0.30	0.10	0.22
2	10	uniform	manhattan	2	1.00	1.00	0.86	0.77	0.97	0.30	0.10	0.22
2	10	distance	minkowski	1	1.00	1.00	0.86	0.77	0.97	0.30	0.10	0.22
2	10	distance	manhattan	1	1.00	1.00	0.86	0.77	0.97	0.30	0.10	0.22
2	10	distance	manhattan	2	1.00	1.00	0.86	0.77	0.97	0.30	0.10	0.22
1	2	uniform	minkowski	1	1.00	1.00	0.86	0.77	0.97	0.30	0.10	0.22
1	2	uniform	manhattan	1	1.00	1.00	0.86	0.77	0.97	0.30	0.10	0.22
1	2	uniform	manhattan	2	1.00	1.00	0.86	0.77	0.97	0.30	0.10	0.22
1	2	distance	minkowski	1	1.00	1.00	0.86	0.77	0.97	0.30	0.10	0.22
1	2	distance	manhattan	1	1.00	1.00	0.86	0.77	0.97	0.30	0.10	0.22
1	2	distance	manhattan	2	1.00	1.00	0.86	0.77	0.97	0.30	0.10	0.22
1	5	uniform	minkowski	1	1.00	1.00	0.86	0.77	0.97	0.30	0.10	0.22
1	5	uniform	manhattan	1	1.00	1.00	0.86	0.77	0.97	0.30	0.10	0.22
1	5	uniform	manhattan	2	1.00	1.00	0.86	0.77	0.97	0.30	0.10	0.22
1	5	distance	minkowski	1	1.00	1.00	0.86	0.77	0.97	0.30	0.10	0.22
1	5	distance	manhattan	1	1.00	1.00	0.86	0.77	0.97	0.30	0.10	0.22
1	5	distance	manhattan	2	1.00	1.00	0.86	0.77	0.97	0.30	0.10	0.22

1	10	uniform	minkowski	1	1.00	1.00	0.86	0.77	0.97	0.30	0.10	0.22
1	10	uniform	manhattan	1	1.00	1.00	0.86	0.77	0.97	0.30	0.10	0.22
1	10	uniform	manhattan	2	1.00	1.00	0.86	0.77	0.97	0.30	0.10	0.22
1	10	distance	minkowski	1	1.00	1.00	0.86	0.77	0.97	0.30	0.10	0.22
1	10	distance	manhattan	1	1.00	1.00	0.86	0.77	0.97	0.30	0.10	0.22
1	10	distance	manhattan	2	1.00	1.00	0.86	0.77	0.97	0.30	0.10	0.22
1	1	uniform	minkowski	1	1.00	1.00	0.86	0.77	0.97	0.30	0.10	0.22
1	1	uniform	manhattan	1	1.00	1.00	0.86	0.77	0.97	0.30	0.10	0.22
1	1	uniform	manhattan	2	1.00	1.00	0.86	0.77	0.97	0.30	0.10	0.22
1	1	distance	minkowski	1	1.00	1.00	0.86	0.77	0.97	0.30	0.10	0.22
1	1	distance	manhattan	1	1.00	1.00	0.86	0.77	0.97	0.30	0.10	0.22
1	1	distance	manhattan	2	1.00	1.00	0.86	0.77	0.97	0.30	0.10	0.22
60	200	uniform	minkowski	2	0.91	0.82	0.90	0.79	0.90	0.59	0.23	0.55
60	30	uniform	minkowski	2	0.91	0.82	0.90	0.79	0.90	0.59	0.23	0.55
60	1	uniform	minkowski	2	0.91	0.82	0.90	0.79	0.90	0.59	0.23	0.55
60	400	uniform	minkowski	2	0.91	0.82	0.90	0.79	0.90	0.59	0.23	0.55
60	200	uniform	minkowski	1	0.92	0.83	0.91	0.81	0.90	0.58	0.22	0.53
60	200	uniform	manhattan	1	0.92	0.83	0.91	0.81	0.90	0.58	0.22	0.53
60	200	uniform	manhattan	2	0.92	0.83	0.91	0.81	0.90	0.58	0.22	0.53
60	30	uniform	minkowski	1	0.92	0.83	0.91	0.81	0.90	0.58	0.22	0.53
60	30	uniform	manhattan	1	0.92	0.83	0.91	0.81	0.90	0.58	0.22	0.53
60	30	uniform	manhattan	2	0.92	0.83	0.91	0.81	0.90	0.58	0.22	0.53
60	400	uniform	minkowski	1	0.92	0.83	0.91	0.81	0.90	0.58	0.22	0.53
60	400	uniform	manhattan	1	0.92	0.83	0.91	0.81	0.90	0.58	0.22	0.53
60	400	uniform	manhattan	2	0.92	0.83	0.91	0.81	0.90	0.58	0.22	0.53
60	1	uniform	minkowski	1	0.92	0.83	0.91	0.81	0.90	0.58	0.22	0.53
60	1	uniform	manhattan	1	0.92	0.83	0.91	0.81	0.90	0.58	0.22	0.53
60	1	uniform	manhattan	2	0.92	0.83	0.91	0.81	0.90	0.58	0.22	0.53
15	200	uniform	minkowski	2	0.94	0.88	0.91	0.82	0.92	0.56	0.20	0.52
15	400	uniform	minkowski	2	0.94	0.88	0.91	0.82	0.92	0.56	0.20	0.52
15	30	uniform	minkowski	2	0.94	0.88	0.91	0.82	0.92	0.56	0.20	0.52
15	1	uniform	minkowski	2	0.94	0.88	0.91	0.82	0.92	0.56	0.20	0.52
40	1	uniform	minkowski	2	0.92	0.84	0.90	0.80	0.90	0.56	0.20	0.52
40	400	uniform	minkowski	2	0.92	0.84	0.90	0.80	0.90	0.56	0.20	0.52
40	30	uniform	minkowski	2	0.92	0.84	0.90	0.80	0.90	0.56	0.20	0.52
40	200	uniform	minkowski	2	0.92	0.84	0.90	0.80	0.90	0.56	0.20	0.52
40	200	uniform	minkowski	1	0.93	0.85	0.91	0.82	0.91	0.56	0.20	0.52
40	200	uniform	manhattan	1	0.93	0.85	0.91	0.82	0.91	0.56	0.20	0.52
40	200	uniform	manhattan	2	0.93	0.85	0.91	0.82	0.91	0.56	0.20	0.52
40	1	uniform	minkowski	1	0.93	0.85	0.91	0.82	0.91	0.56	0.20	0.52
40	1	uniform	manhattan	1	0.93	0.85	0.91	0.82	0.91	0.56	0.20	0.52
40	1	uniform	manhattan	2	0.93	0.85	0.91	0.82	0.91	0.56	0.20	0.52
40	30	uniform	minkowski	1	0.93	0.85	0.91	0.82	0.91	0.56	0.20	0.52
40	30	uniform	manhattan	1	0.93	0.85	0.91	0.82	0.91	0.56	0.20	0.52
40	30	uniform	manhattan	2	0.93	0.85	0.91	0.82	0.91	0.56	0.20	0.52
40	400	uniform	minkowski	1	0.93	0.85	0.91	0.82	0.91	0.56	0.20	0.52

40	400	uniform	manhattan	1	0.93	0.85	0.91	0.82	0.91	0.56	0.20	0.52
40	400	uniform	manhattan	2	0.93	0.85	0.91	0.82	0.91	0.56	0.20	0.52
15	1	uniform	minkowski	1	0.95	0.89	0.92	0.84	0.92	0.56	0.20	0.52
15	1	uniform	manhattan	1	0.95	0.89	0.92	0.84	0.92	0.56	0.20	0.52
15	1	uniform	manhattan	2	0.95	0.89	0.92	0.84	0.92	0.56	0.20	0.52
15	400	uniform	minkowski	1	0.95	0.89	0.92	0.84	0.92	0.56	0.20	0.52
15	400	uniform	manhattan	1	0.95	0.89	0.92	0.84	0.92	0.56	0.20	0.52
15	400	uniform	manhattan	2	0.95	0.89	0.92	0.84	0.92	0.56	0.20	0.52
15	30	uniform	minkowski	1	0.95	0.89	0.92	0.84	0.92	0.56	0.20	0.52
15	30	uniform	manhattan	1	0.95	0.89	0.92	0.84	0.92	0.56	0.20	0.52
15	30	uniform	manhattan	2	0.95	0.89	0.92	0.84	0.92	0.56	0.20	0.52
15	200	uniform	minkowski	1	0.95	0.89	0.92	0.84	0.92	0.56	0.20	0.52
15	200	uniform	manhattan	1	0.95	0.89	0.92	0.84	0.92	0.56	0.20	0.52
15	200	uniform	manhattan	2	0.95	0.89	0.92	0.84	0.92	0.56	0.20	0.52
25	1	uniform	minkowski	1	0.94	0.87	0.92	0.83	0.91	0.54	0.19	0.50
25	1	uniform	manhattan	1	0.94	0.87	0.92	0.83	0.91	0.54	0.19	0.50
25	1	uniform	manhattan	2	0.94	0.87	0.92	0.83	0.91	0.54	0.19	0.50
25	200	uniform	minkowski	1	0.94	0.87	0.92	0.83	0.91	0.54	0.19	0.50
25	200	uniform	manhattan	1	0.94	0.87	0.92	0.83	0.91	0.54	0.19	0.50
25	200	uniform	manhattan	2	0.94	0.87	0.92	0.83	0.91	0.54	0.19	0.50
25	400	uniform	minkowski	1	0.94	0.87	0.92	0.83	0.91	0.54	0.19	0.50
25	400	uniform	manhattan	1	0.94	0.87	0.92	0.83	0.91	0.54	0.19	0.50
25	400	uniform	manhattan	2	0.94	0.87	0.92	0.83	0.91	0.54	0.19	0.50
25	30	uniform	minkowski	1	0.94	0.87	0.92	0.83	0.91	0.54	0.19	0.50
25	30	uniform	manhattan	1	0.94	0.87	0.92	0.83	0.91	0.54	0.19	0.50
25	30	uniform	manhattan	2	0.94	0.87	0.92	0.83	0.91	0.54	0.19	0.50
25	400	uniform	minkowski	2	0.93	0.86	0.91	0.81	0.91	0.54	0.19	0.50
25	1	uniform	minkowski	2	0.93	0.86	0.91	0.81	0.91	0.54	0.19	0.50
25	30	uniform	minkowski	2	0.93	0.86	0.91	0.81	0.91	0.54	0.19	0.50
25	200	uniform	minkowski	2	0.93	0.86	0.91	0.81	0.91	0.54	0.19	0.50
10	200	uniform	minkowski	2	0.96	0.92	0.91	0.82	0.93	0.50	0.17	0.45
10	400	uniform	minkowski	2	0.96	0.92	0.91	0.82	0.93	0.50	0.17	0.45
10	1	uniform	minkowski	2	0.96	0.92	0.91	0.82	0.93	0.50	0.17	0.45
10	30	uniform	minkowski	2	0.96	0.92	0.91	0.82	0.93	0.50	0.17	0.45
10	1	uniform	minkowski	1	0.96	0.93	0.92	0.85	0.93	0.51	0.17	0.45
10	1	uniform	manhattan	1	0.96	0.93	0.92	0.85	0.93	0.51	0.17	0.45
10	1	uniform	manhattan	2	0.96	0.93	0.92	0.85	0.93	0.51	0.17	0.45
10	200	uniform	minkowski	1	0.96	0.93	0.92	0.85	0.93	0.51	0.17	0.45
10	200	uniform	manhattan	1	0.96	0.93	0.92	0.85	0.93	0.51	0.17	0.45
10	200	uniform	manhattan	2	0.96	0.93	0.92	0.85	0.93	0.51	0.17	0.45
10	30	uniform	minkowski	1	0.96	0.93	0.92	0.85	0.93	0.51	0.17	0.45
10	30	uniform	manhattan	1	0.96	0.93	0.92	0.85	0.93	0.51	0.17	0.45
10	30	uniform	manhattan	2	0.96	0.93	0.92	0.85	0.93	0.51	0.17	0.45
10	400	uniform	minkowski	1	0.96	0.93	0.92	0.85	0.93	0.51	0.17	0.45
10	400	uniform	manhattan	1	0.96	0.93	0.92	0.85	0.93	0.51	0.17	0.45
10	400	uniform	manhattan	2	0.96	0.93	0.92	0.85	0.93	0.51	0.17	0.45

55	250	uniform	minkowski	2	0.91	0.82	0.90	0.80	0.90	0.60	0.23	0.57
55	150	uniform	minkowski	2	0.91	0.82	0.90	0.80	0.90	0.60	0.23	0.57
55	200	uniform	minkowski	2	0.91	0.82	0.90	0.80	0.90	0.60	0.23	0.57
50	150	uniform	minkowski	2	0.92	0.83	0.90	0.80	0.90	0.59	0.22	0.56
50	200	uniform	minkowski	2	0.92	0.83	0.90	0.80	0.90	0.59	0.22	0.56
50	250	uniform	minkowski	2	0.92	0.83	0.90	0.80	0.90	0.59	0.22	0.56
60	150	uniform	minkowski	2	0.91	0.82	0.90	0.79	0.90	0.59	0.23	0.55
60	200	uniform	minkowski	2	0.91	0.82	0.90	0.79	0.90	0.59	0.23	0.55
60	250	uniform	minkowski	2	0.91	0.82	0.90	0.79	0.90	0.59	0.23	0.55
45	150	uniform	minkowski	2	0.92	0.83	0.90	0.80	0.90	0.58	0.21	0.54
45	200	uniform	minkowski	2	0.92	0.83	0.90	0.80	0.90	0.58	0.21	0.54
45	250	uniform	minkowski	2	0.92	0.83	0.90	0.80	0.90	0.58	0.21	0.54
40	250	uniform	minkowski	2	0.92	0.84	0.90	0.80	0.90	0.56	0.20	0.52
40	150	uniform	minkowski	2	0.92	0.84	0.90	0.80	0.90	0.56	0.20	0.52
40	200	uniform	minkowski	2	0.92	0.84	0.90	0.80	0.90	0.56	0.20	0.52
59	250	uniform	minkowski	2	0.91	0.82	0.90	0.79	0.90	0.61	0.23	0.58
59	300	uniform	minkowski	2	0.91	0.82	0.90	0.79	0.90	0.61	0.23	0.58
59	200	uniform	minkowski	2	0.91	0.82	0.90	0.79	0.90	0.61	0.23	0.58
58	250	uniform	minkowski	2	0.91	0.82	0.90	0.80	0.90	0.60	0.23	0.58
58	300	uniform	minkowski	2	0.91	0.82	0.90	0.80	0.90	0.60	0.23	0.58
57	250	uniform	minkowski	2	0.91	0.82	0.90	0.80	0.90	0.60	0.23	0.58
57	300	uniform	minkowski	2	0.91	0.82	0.90	0.80	0.90	0.60	0.23	0.58
58	200	uniform	minkowski	2	0.91	0.82	0.90	0.80	0.90	0.60	0.23	0.58
57	200	uniform	minkowski	2	0.91	0.82	0.90	0.80	0.90	0.60	0.23	0.58
56	250	uniform	minkowski	2	0.91	0.82	0.90	0.80	0.90	0.60	0.23	0.57
56	300	uniform	minkowski	2	0.91	0.82	0.90	0.80	0.90	0.60	0.23	0.57
56	200	uniform	minkowski	2	0.91	0.82	0.90	0.80	0.90	0.60	0.23	0.57
55	250	uniform	minkowski	2	0.91	0.82	0.90	0.80	0.90	0.60	0.23	0.57
55	300	uniform	minkowski	2	0.91	0.82	0.90	0.80	0.90	0.60	0.23	0.57
55	200	uniform	minkowski	2	0.91	0.82	0.90	0.80	0.90	0.60	0.23	0.57
54	200	uniform	minkowski	2	0.91	0.82	0.90	0.80	0.90	0.60	0.23	0.57
54	250	uniform	minkowski	2	0.91	0.82	0.90	0.80	0.90	0.60	0.23	0.57
54	300	uniform	minkowski	2	0.91	0.82	0.90	0.80	0.90	0.60	0.23	0.57
53	200	uniform	minkowski	2	0.91	0.82	0.90	0.80	0.90	0.60	0.23	0.57
53	250	uniform	minkowski	2	0.91	0.82	0.90	0.80	0.90	0.60	0.23	0.57
53	300	uniform	minkowski	2	0.91	0.82	0.90	0.80	0.90	0.60	0.23	0.57
52	200	uniform	minkowski	2	0.91	0.82	0.90	0.80	0.90	0.59	0.22	0.56
52	250	uniform	minkowski	2	0.91	0.82	0.90	0.80	0.90	0.59	0.22	0.56
52	300	uniform	minkowski	2	0.91	0.82	0.90	0.80	0.90	0.59	0.22	0.56
51	200	uniform	minkowski	2	0.91	0.82	0.90	0.80	0.90	0.59	0.22	0.56
51	250	uniform	minkowski	2	0.91	0.82	0.90	0.80	0.90	0.59	0.22	0.56
51	300	uniform	minkowski	2	0.91	0.82	0.90	0.80	0.90	0.59	0.22	0.56
59	300	uniform	minkowski	2	0.91	0.82	0.90	0.79	0.90	0.61	0.23	0.58
59	400	uniform	minkowski	2	0.91	0.82	0.90	0.79	0.90	0.61	0.23	0.58
65	300	uniform	minkowski	2	0.91	0.82	0.90	0.79	0.90	0.60	0.23	0.56
65	400	uniform	minkowski	2	0.91	0.82	0.90	0.79	0.90	0.60	0.23	0.56

64	300	uniform	minkowski	2	0.91	0.82	0.90	0.79	0.90	0.59	0.23	0.56
64	400	uniform	minkowski	2	0.91	0.82	0.90	0.79	0.90	0.59	0.23	0.56
63	300	uniform	minkowski	2	0.91	0.82	0.90	0.79	0.90	0.59	0.23	0.56
63	400	uniform	minkowski	2	0.91	0.82	0.90	0.79	0.90	0.59	0.23	0.56
61	300	uniform	minkowski	2	0.91	0.82	0.90	0.79	0.90	0.59	0.23	0.55
61	400	uniform	minkowski	2	0.91	0.82	0.90	0.79	0.90	0.59	0.23	0.55
62	300	uniform	minkowski	2	0.91	0.82	0.90	0.79	0.90	0.59	0.23	0.55
62	400	uniform	minkowski	2	0.91	0.82	0.90	0.79	0.90	0.59	0.23	0.55
60	300	uniform	minkowski	2	0.91	0.82	0.90	0.79	0.90	0.59	0.23	0.55
60	400	uniform	minkowski	2	0.91	0.82	0.90	0.79	0.90	0.59	0.23	0.55
75	300	uniform	minkowski	2	0.91	0.81	0.90	0.79	0.89	0.60	0.23	0.59
59	300	uniform	minkowski	2	0.91	0.82	0.90	0.79	0.90	0.61	0.23	0.58
73	300	uniform	minkowski	2	0.91	0.81	0.90	0.79	0.90	0.60	0.23	0.58
74	300	uniform	minkowski	2	0.91	0.81	0.90	0.79	0.90	0.60	0.23	0.58
71	300	uniform	minkowski	2	0.91	0.81	0.90	0.79	0.90	0.60	0.23	0.58
72	300	uniform	minkowski	2	0.91	0.81	0.90	0.79	0.90	0.60	0.23	0.58
70	300	uniform	minkowski	2	0.91	0.81	0.90	0.79	0.90	0.60	0.23	0.57
69	300	uniform	minkowski	2	0.91	0.81	0.90	0.79	0.90	0.60	0.23	0.57
68	300	uniform	minkowski	2	0.91	0.82	0.90	0.79	0.90	0.60	0.23	0.57
67	300	uniform	minkowski	2	0.91	0.82	0.90	0.79	0.90	0.60	0.23	0.57
66	300	uniform	minkowski	2	0.91	0.82	0.90	0.79	0.90	0.60	0.23	0.56
79	300	uniform	minkowski	2	0.91	0.81	0.90	0.79	0.89	0.61	0.23	0.59
78	300	uniform	minkowski	2	0.91	0.81	0.90	0.79	0.89	0.60	0.23	0.59
77	300	uniform	minkowski	2	0.91	0.81	0.90	0.79	0.89	0.60	0.23	0.59
75	300	uniform	minkowski	2	0.91	0.81	0.90	0.79	0.89	0.60	0.23	0.59
76	300	uniform	minkowski	2	0.91	0.81	0.90	0.79	0.89	0.60	0.23	0.59
59	300	uniform	minkowski	2	0.91	0.82	0.90	0.79	0.90	0.61	0.23	0.58
85	300	uniform	minkowski	2	0.91	0.81	0.90	0.79	0.89	0.60	0.22	0.58
84	300	uniform	minkowski	2	0.91	0.81	0.90	0.79	0.89	0.59	0.22	0.57
83	300	uniform	minkowski	2	0.91	0.81	0.90	0.79	0.89	0.59	0.22	0.57
82	300	uniform	minkowski	2	0.91	0.81	0.90	0.79	0.89	0.59	0.22	0.57
81	300	uniform	minkowski	2	0.91	0.81	0.90	0.79	0.89	0.59	0.22	0.57
80	300	uniform	minkowski	2	0.91	0.81	0.90	0.79	0.89	0.59	0.22	0.56
390	300	uniform	minkowski	2	0.89	0.77	0.89	0.77	0.88	0.62	0.23	0.63
370	300	uniform	minkowski	2	0.89	0.78	0.89	0.77	0.88	0.62	0.23	0.62
400	300	uniform	minkowski	2	0.89	0.77	0.89	0.77	0.88	0.62	0.23	0.62
350	300	uniform	minkowski	2	0.89	0.78	0.89	0.77	0.88	0.62	0.23	0.62
316	300	uniform	minkowski	2	0.89	0.78	0.89	0.77	0.89	0.61	0.23	0.62
380	300	uniform	minkowski	2	0.89	0.78	0.89	0.77	0.88	0.62	0.23	0.62
360	300	uniform	minkowski	2	0.89	0.78	0.89	0.77	0.88	0.62	0.23	0.62
237	300	uniform	minkowski	2	0.89	0.78	0.89	0.77	0.89	0.61	0.22	0.62
158	300	uniform	minkowski	2	0.90	0.79	0.89	0.78	0.89	0.61	0.22	0.61
79	300	uniform	minkowski	2	0.91	0.81	0.90	0.79	0.89	0.61	0.23	0.59
399	300	uniform	minkowski	2	0.89	0.77	0.89	0.77	0.88	0.62	0.23	0.63
398	300	uniform	minkowski	2	0.89	0.77	0.89	0.77	0.88	0.62	0.23	0.63
397	300	uniform	minkowski	2	0.89	0.77	0.89	0.77	0.88	0.62	0.23	0.63

396	300	uniform	minkowski	2	0.89	0.77	0.89	0.77	0.88	0.62	0.23	0.63
395	300	uniform	minkowski	2	0.89	0.77	0.89	0.77	0.88	0.62	0.23	0.63
394	300	uniform	minkowski	2	0.89	0.77	0.89	0.77	0.88	0.62	0.23	0.63
393	300	uniform	minkowski	2	0.89	0.77	0.89	0.77	0.88	0.62	0.23	0.63
392	300	uniform	minkowski	2	0.89	0.77	0.89	0.77	0.88	0.62	0.23	0.63
391	300	uniform	minkowski	2	0.89	0.77	0.89	0.77	0.88	0.62	0.23	0.63
390	300	uniform	minkowski	2	0.89	0.77	0.89	0.77	0.88	0.62	0.23	0.63
335	300	uniform	minkowski	2	0.89	0.78	0.89	0.77	0.88	0.65	0.30	0.59
385	300	uniform	minkowski	2	0.89	0.77	0.89	0.77	0.88	0.65	0.30	0.59
285	300	uniform	minkowski	2	0.89	0.78	0.89	0.77	0.89	0.65	0.30	0.58
235	300	uniform	minkowski	2	0.89	0.78	0.89	0.77	0.89	0.65	0.30	0.58
185	300	uniform	minkowski	2	0.90	0.79	0.89	0.78	0.89	0.64	0.30	0.57
135	300	uniform	minkowski	2	0.90	0.79	0.89	0.78	0.89	0.64	0.29	0.56
85	300	uniform	minkowski	2	0.90	0.80	0.90	0.79	0.89	0.62	0.28	0.52
35	300	uniform	minkowski	2	0.92	0.83	0.90	0.80	0.90	0.61	0.27	0.50
385	300	uniform	minkowski	2	0.90	0.79	0.89	0.79	0.89	0.65	0.31	0.54
335	300	uniform	minkowski	2	0.90	0.79	0.89	0.79	0.89	0.65	0.31	0.54
285	300	uniform	minkowski	2	0.90	0.79	0.90	0.79	0.89	0.65	0.31	0.53
235	300	uniform	minkowski	2	0.90	0.79	0.90	0.79	0.89	0.65	0.31	0.52
185	300	uniform	minkowski	2	0.90	0.79	0.90	0.79	0.89	0.65	0.31	0.50
135	300	uniform	minkowski	2	0.90	0.80	0.90	0.79	0.90	0.65	0.31	0.48
85	300	uniform	minkowski	2	0.90	0.80	0.90	0.79	0.90	0.63	0.31	0.45
35	300	uniform	minkowski	2	0.91	0.82	0.90	0.79	0.90	0.60	0.29	0.40
395	300	uniform	minkowski	2	0.89	0.77	0.89	0.77	0.88	0.65	0.30	0.60
375	300	uniform	minkowski	2	0.89	0.77	0.89	0.77	0.88	0.65	0.30	0.59
355	300	uniform	minkowski	2	0.89	0.77	0.89	0.77	0.88	0.65	0.30	0.59
335	300	uniform	minkowski	2	0.89	0.78	0.89	0.77	0.88	0.65	0.30	0.59
385	300	uniform	minkowski	2	0.89	0.77	0.89	0.77	0.88	0.65	0.30	0.59
315	300	uniform	minkowski	2	0.89	0.78	0.89	0.77	0.89	0.65	0.30	0.59
365	300	uniform	minkowski	2	0.89	0.77	0.89	0.77	0.88	0.65	0.30	0.59
345	300	uniform	minkowski	2	0.89	0.78	0.89	0.77	0.88	0.65	0.30	0.59
295	300	uniform	minkowski	2	0.89	0.78	0.89	0.77	0.89	0.65	0.30	0.59
325	300	uniform	minkowski	2	0.89	0.78	0.89	0.77	0.88	0.65	0.30	0.59
305	300	uniform	minkowski	2	0.89	0.78	0.89	0.77	0.89	0.65	0.30	0.59
395	300	uniform	minkowski	2	0.90	0.79	0.89	0.78	0.89	0.66	0.31	0.55
385	300	uniform	minkowski	2	0.90	0.79	0.89	0.79	0.89	0.65	0.31	0.54
375	300	uniform	minkowski	2	0.90	0.79	0.89	0.79	0.89	0.65	0.30	0.54
355	300	uniform	minkowski	2	0.90	0.79	0.89	0.79	0.89	0.65	0.30	0.54
365	300	uniform	minkowski	2	0.90	0.79	0.89	0.79	0.89	0.65	0.30	0.54
335	300	uniform	minkowski	2	0.90	0.79	0.89	0.79	0.89	0.65	0.31	0.54
345	300	uniform	minkowski	2	0.90	0.79	0.89	0.79	0.89	0.65	0.31	0.54
315	300	uniform	minkowski	2	0.90	0.79	0.90	0.79	0.89	0.65	0.30	0.53
325	300	uniform	minkowski	2	0.90	0.79	0.90	0.79	0.89	0.65	0.31	0.53
295	300	uniform	minkowski	2	0.90	0.79	0.90	0.79	0.89	0.65	0.30	0.53
305	300	uniform	minkowski	2	0.90	0.79	0.90	0.79	0.89	0.65	0.30	0.53
399	300	uniform	minkowski	2	0.89	0.77	0.89	0.77	0.88	0.65	0.30	0.60

397	300	uniform	minkowski	2	0.89	0.77	0.89	0.77	0.88	0.65	0.30	0.60
395	300	uniform	minkowski	2	0.89	0.77	0.89	0.77	0.88	0.65	0.30	0.60
393	300	uniform	minkowski	2	0.89	0.77	0.89	0.77	0.88	0.65	0.30	0.60
391	300	uniform	minkowski	2	0.89	0.77	0.89	0.77	0.88	0.65	0.30	0.59
389	300	uniform	minkowski	2	0.89	0.77	0.89	0.77	0.88	0.65	0.30	0.59
387	300	uniform	minkowski	2	0.89	0.77	0.89	0.77	0.88	0.65	0.30	0.59
385	300	uniform	minkowski	2	0.89	0.77	0.89	0.77	0.88	0.65	0.30	0.59
383	300	uniform	minkowski	2	0.89	0.77	0.89	0.77	0.88	0.65	0.29	0.59
381	300	uniform	minkowski	2	0.89	0.77	0.89	0.77	0.88	0.65	0.30	0.59
379	300	uniform	minkowski	2	0.89	0.77	0.89	0.77	0.88	0.65	0.30	0.60
377	300	uniform	minkowski	2	0.89	0.77	0.89	0.77	0.88	0.65	0.30	0.60
399	300	uniform	minkowski	2	0.90	0.79	0.89	0.78	0.89	0.66	0.31	0.55
397	300	uniform	minkowski	2	0.90	0.79	0.89	0.78	0.89	0.66	0.31	0.55
395	300	uniform	minkowski	2	0.90	0.79	0.89	0.78	0.89	0.66	0.31	0.55
393	300	uniform	minkowski	2	0.90	0.79	0.89	0.78	0.89	0.66	0.31	0.55
391	300	uniform	minkowski	2	0.90	0.79	0.89	0.78	0.89	0.65	0.31	0.54
389	300	uniform	minkowski	2	0.90	0.79	0.89	0.78	0.89	0.65	0.31	0.54
379	300	uniform	minkowski	2	0.90	0.79	0.89	0.78	0.89	0.65	0.30	0.54
377	300	uniform	minkowski	2	0.90	0.79	0.89	0.79	0.89	0.65	0.30	0.54
387	300	uniform	minkowski	2	0.90	0.79	0.89	0.79	0.89	0.65	0.31	0.54
385	300	uniform	minkowski	2	0.90	0.79	0.89	0.79	0.89	0.65	0.31	0.54
383	300	uniform	minkowski	2	0.90	0.79	0.89	0.79	0.89	0.65	0.30	0.54
381	300	uniform	minkowski	2	0.90	0.79	0.89	0.79	0.89	0.65	0.30	0.54
